# Evidence-Based Structural Model of the Staphylococcal Repressor Protein: Separation of Functions into Different Domains

**DOI:** 10.1371/journal.pone.0139086

**Published:** 2015-09-28

**Authors:** Kinga Nyíri, Bianka Kőhegyi, András Micsonai, József Kardos, Beata G. Vertessy

**Affiliations:** 1 Department of Applied Biotechnology and Food Sciences, Budapest University of Technology and Economics, Budapest, 1111, Hungary; 2 Institute of Enzymology, Research Centre for Natural Sciences, Hungarian Academy of Sciences, Budapest, 1117, Hungary; 3 Department of Biochemistry and MTA-ELTE NAP B Neuroimmunology Research Group, Institute of Biology, Eötvös Loránd University, Budapest, 1117, Hungary; Russian Academy of Sciences, Institute for Biological Instrumentation, RUSSIAN FEDERATION

## Abstract

Horizontal transfer of mobile genetic elements within *Staphylococci* is of high biomedical significance as such elements are frequently responsible for virulence and toxic effects. *Staphylococcus*-encoded repressor proteins regulate the replication of these mobile genetic elements that are located within the so-called pathogenicity islands. Here, we report structural and functional characterization of one such repressor protein, namely the Stl protein encoded by the pathogenicity island SaPIbov1. We create a 3D structural model and based on this prediction, we investigate the different functionalities of truncated and point mutant constructs. Results suggest that a helix-turn-helix motif governs the interaction of the Stl protein with its cognate DNA site: point mutations within this motif drastically decrease DNA-binding ability, whereas the interaction with the Stl-binding partner protein dUTPase is unperturbed by these point mutations. The 3D model also suggested the potential independent folding of a carboxy-terminal domain. This suggestion was fully verified by independent experiments revealing that the carboxy-terminal domain does not bind to DNA but is still capable of binding to and inhibiting dUTPase. A general model is proposed, which suggests that among the several structurally different repressor superfamilies Stl-like Staphylococcal repressor proteins belong to the helix-turn-helix transcription factor group and the HTH motif is suggested to reside within N-terminal segment.

## Introduction

Phage mediated mobilization of pathogenicity islands, i.e. genetic elements encoding virulence factors and toxins in *Staphylococcus aureus* (SaPI) has been an intensively studied field in recent years [1]. It has been shown that excision and replication of SaPIs is induced by formation of a repressor:derepressor complex constituting the Staphylococcal master repressor protein and another phage-related protein [2,3]. In the specific case of the SaPIbov1 pathogenicity island repressor Stl (abbreviated as Stl in the present study), the derepressor is a phage-related dUTPase enzyme [3]. dUTPases are important guardians of genome integrity [4,5]. Their physiological role is to deplete the cellular dUTP pool to prevent incorporation of uracil into DNA [6,7]. The enzyme family of dUTPases constitutes two large subfamilies with different protein structure but very similar catalytic function [8,9].

In-depth studies on the Stl:dUTPase interaction revealed an additional function of the Stl repressor, namely it has been proven to be an effective inhibitor of the dUTPase enzyme from the Φ11 Staphylococcal phage [10]. In addition, we have recently shown that Stl might be a cross-species general dUTPase inhibitor, which may open new horizons in studying dUTPase cellular function [11]. Moreover, since dUTPase has been proposed as a significant novel target in antimycobacterial drug design [12–14], Stl may also be a possible candidate for designing protein-based dUTPase-inhibitors to fight *Mycobacterium tuberculosis*.

For the dUTPase enzyme family, detailed studies have already addressed its structural and functional characteristics [8,15–21]. In contrast to the essential role of Stl-like repressors in the patho-mechanism and in the horizontal gene transfer of several toxins, no structural information has yet been reported for any of the SaPI repressor proteins or their complexes.

The SaPIs are generally considered as phage originated genetic elements, and this is supported by their mobilization by phage proteins. Similarly to master repressors of temperate phages Stl is an autoinducer [2], and blocks the expression of genes *int*, *xis* and *str*, which are responsible for SaPI excision and replication [22,23]. It has been shown that one of the DNA-binding sites of Stl resides within the *stl*-*str* intergenic region, which coincides with the repression of genes downstream to that region [22]. Other binding sites or sequence specificity of Stl have not been identified yet, however the question is intensively studied by our laboratory.

Based on functional similarities, the gene regulation mechanism of Stl may be adequately modeled by the mechanism of the main lifecycle regulator CI repressor of temperate phages. Within this model, repressor proteins are responsible for binding to a specific DNA segment thereby preventing excision and replication of the relevant genetic segments. The CI repressor proteins have separate domains for DNA binding and protein binding [24,25]. The protein binding domain is usually responsible for oligomerization of the repressor and for the interaction with the derepressor protein [24,26–28]. The oligomerization makes the regulation more sensitive to the alteration of protein concentration since it depends on the oligomer monomer equilibrium [28]. Higher order oligomers are suitable for more complex regulation patterns [29,30]. The mechanism of derepression can be reversible or irreversible. Prophage induction in lambdoid phages is achieved through a RecA binding induced specific autoproteolytic inactivation of the maintenance repressor [31]. Several P2 related repressors are inactivated through a noncovalent complex formation with the derepressor protein [32–35]. The DNA binding function of repressors can be performed through several different structural motifs, such as helix-turn-helix (HTH) as in CI repressor protein of lambda phage, winged- helix turn helix (wHTH) as in the MuR repressor protein of Mu phage, and the antiparallel β-strands of the ribbon-helix-helix (RHH) fold as in Arc repressor of P22 phage [36,37].

Based on these models, our focus was to resolve how potential domains within the Stl repressor may be defined. Towards this end, first we created a structural model of the full length Stl protein and investigated its folding by synchrotron radiation circular dichroism (SRCD) measurements. Based on the 3D model verified by CD results, we produced truncated and point mutants and studied their function in DNA and dUTPase binding. We show that the produced carboxy-terminal segment is an independently folded domain, which retains its binding affinity to dUTPase, but shows reduced inhibitory effect. The amino-terminal putatively DNA binding segment was also studied by point mutations. Our experimental results convincingly support the predicted position of helix-turn-helix motif.

## Materials and Methods

### Homology modeling and *in silico* predictions

The 3D homology model of Stl was constructed using the Phyre2 Server in intensive mode. Seven templates were selected by the program (PDB IDs: 1E3O, 4YV9, 2GRM, 4RYK, 2QFC, 2AXZ, 2EBY) to model Stl protein based on heuristics to maximize confidence, percentage identity and alignment coverage [38]. Five out of the seven templates covered >90% of the Stl sequence, while the other two templates provided only partial coverage of the sequence, but with higher local similarity. In the final model, 97% of residues were modeled at >90% confidence (see Table A in S1 File for additional information on the template proteins). Homology prediction was made using HHpred [39], subsequent 3D structure predictions with Modeller was performed with automatic template selection and also with the Phyre2 hits manually selected as templates from the HHpred list [40]. Related structures were searched in the Molecular Modeling Database (MMDB) also referred to as the Entrez Structure database [41]. Functional domain search was performed by Pfam and NCBI Conserved Domain Database [42,43]. The possible position of the helix-turn-helix (HTH) DNA binding motif in the sequence was predicted by NPS@ server [44]. Disorder prediction during construct design was performed by using GeneSilico MetaDisorder service [45]. To compare the homology model to the structural information obtained experimentally by SRCD spectroscopy, the secondary structure composition of the model, including the helix content, was assigned using the DSSP algorithm [46] and the BeStSel and CONTIN secondary structure definitions [47,48].

### Cloning and expression of proteins

The cDNA of Stl-CTD was made by PCR amplification from the pGEX-4T-1 vector containing Stl protein (GenBank ID AAG29617.1) described in our previous work [10]. For the amplification of Stl-CTD the Stl-CTD-F (5’-TATTGAATTCAGCCCGACCCTGAACG-3’) and the Stl-CTD-R (5’-GGTCCTCGAGTT AGTTGGTATCTTTTTCCAGAATAATTTTTTTCTGATG-3’) primers were used. The resulting insert was cloned with EcoRI and XhoI restriction sites of the pGEX-4T-1 vector in frame with the amino-terminal GST tag and the thrombin cleavage site. A stop codon was mutated to the full length sequence to provide a construct for the N-terminal segment of Stl (residues 1–84) by QuikChange site-directed mutagenesis (Stratagene) using mutagenic primers Stl-NT-F 5'- GCGATGAATTTAAAGAAAAAGGCTATTAGCTAACTGAGCCCGACCCT GAACG -3') and Stl-NT-R 5'- CGTTCAGGGTCGGGCTCAGTTAGCTAATAGCCTTTTTCTTTAAATTCAT CGC -3'. Stl- Q40A,N41A (Stl-AA) mutant was created from the original vector by QuikChange site-directed mutagenesis (Stratagene) using mutagenic primers Stl-AA-F 5'-CGTTTTCATGGTTGCTAATGGTCGCT GCGCTAAAGCCGGTGCG-3') and Stl-AA-R 5'-CGCACCGGCTTTAGCGCAGCGACCATTAGCAACCAT GAAAACG-3'. DNA sequencing for verification of the resulting constructs were performed by Eurofins MWG Operon. Vectors were transformed to into *Escherichia coli* strain BL21 Rosetta (DE3) and propagated in 500 ml LB till exponential growth, then the culture was induced with 0.5 mM iso-propyl-β-D-thiogalactoside. After induction, the cell cultures were grown at 303 K for further 4 h. Finally the cells were harvested by centrifugation and stored at 193 K. Protein over-expression from the created constructs, except the one which encodes the N-terminal segment, was successful.

### Purification of proteins

For purification of GST-tagged proteins (Stl, Stl-CTD, Stl-AA), cell pellets were solubilized using Potter-Elvehjem homogenizer in 20 ml buffer A (15 ml Hepes (pH 7.5), 200 mM NaCl) supplemented with 2 mM dithiothreitol (DTT), 1% Triton X-100, ca. 2 μg/ml RNase and DNase and one tablet of Complete ULTRA Tablets, Mini, EDTA-free protease inhibitor. Cell suspensions were sonicated (4 x 60 s), and centrifuged (16000g for 30 min). Supernatant loaded on a pre-equilibrated benchtop glutathione-agarose affinity-chromatography column (GE Healthcare). The column was washed with ten volumes of buffer A (200 mM NaCl). After that 80 Cleavage Units thrombin (GE Healthcare) was added to perform on-column cleavage for the removal of GST tag. After overnight cleavage purified were obtained in the flow-through.

Purification of Φ11dUTPase was performed as described previously [49]. Briefly, supernatant resulting from centrifugation of cell lysate was purified on Q-Sepharose (GE Healthcare) anion-exchange column, followed by gel filtration on a Superdex 75 column (GE Healthcare) using an AKTA Explorer purifier. Protein preparations were used freshly or were flash-frozen in liquid nitrogen, and stored at 193 K. All protein preparations were >95% pure as judged by SDS–PAGE.

### Protein quantification

Protein concentration was measured using NanoDrop 2000 UV-Vis spectrophotometer using the following A ^0.1%^_280_ values 1.087, 1.261, 1.090 and 0.786 ml * mg^-1^* cm^-1^ for Stl, Stl-CTD, Stl-AA and Φ11dUTPase respectively, calculated based on amino acid composition (http://web.expasy.org/protparam/).

### Native polyacrylamide gel electrophoresis

Native gel electrophoresis was performed in 8% polyacrylamide gel. After preparation the gel was subjected to pre-electrophoresis with constant voltages of 100 V. Then 25 μl of the premixed samples was applied on the gel and electrophoresis was performed for 1.5 hours on 150 V in pH 8.7 Tris-HCl buffer. The apparatus was cooled on ice during electrophoresis in order to avoid denaturation caused by the evolving heat. Coomassie-Brilliant Blue dye washed to stain the gel.

### Electrophoretic mobility shift assay (EMSA)

EMSA experiments were performed using a 57mer dsDNA oligonucleotide (5’-GCTCATATTATTCCTCTCCTACCATTTTATCTCTAATTGAGATATTTATATTCAGAT-3’) based on our previous results. Complementary oligonucleotides were custom synthesized by Eurofins MWG Operon and hybridized by controlled gradual cooling after 5 minutes incubation on 95°C. The investigated proteins were mixed with 100 ng DNA and in 20 μl total volume, concentration of NaCl was set to 100 mM in all the samples. After incubation for 15 min at 4°C, samples were loaded onto 8% polyacrylamide gel. Electrophoresis was performed in Tris- Borate- EDTA (TBE) buffer for 70 min at room temperature, following 1 h pre-electrophoresis of the gel. Bands were detected after staining with GelRed (Biotium), using a Uvi-Tec geldocumentation system (Cleaver Scientific Ltd., Rugby, UK).

### Steady-state kinetics experiments

Proton release during the transformation of dUTP into dUMP and PPi was followed using a Jasco V550 spectrophotometer at 559 nm and 293 K [50]. Reaction mixtures contained 20 nM Φ11 dUTPase enzyme in 1 mM HEPES–HCl (pH 7.5) containing 5 mM MgCl_2_, 150 mM KCl and 40 mM phenol red pH indicator. After preincubation of the two proteins for 5 minutes the reaction was started with the addition of 20 mM dUTP. The initial velocity was determined from the slope of the first 10% of the progress curve. Quadratic binding equation was fitted to the data.

### Synchrotron radiation circular dichroism (SRCD) measurements and CD spectrum analysis

SRCD spectrum of Stl was recorded at the DISCO beamline of SOLEIL French Synchrotron Facility (proposal No. 20140646). The Stl concentration was 2.1 mg/ml in a buffer of 50 mM Hepes, 200 mM NaCl, pH 7.5. A CaF_2_ cell with a path length of 6.13 μm was used. 38 scans were accumulated in the 180–270 nm wavelength range at 1 nm steps with a lock-in time constant of 300 msec and integration time of 1200 msec. In this wavelength range and path length, the photomultiplier voltage did not exceed the 700 V limit. After baseline subtraction, the spectrum was corrected with the CSA calibration [51].

To estimate the secondary structure content, the CD spectrum was analyzed by the BeStSel [47] and CONTIN methods [48]. These algorithms distinguish two types of spectrally different helical components, helix1 and helix2. Helix1 is the regular, middle part of the helix where all the backbone-backbone hydrogen bonds are formed and helix2, called “distorted helix”, consists of the two-two residues at the ends of the helix with unsatisfied H-bonding. The helix2 content together with the helix2/helix1 ratio provide a chance to predict the number and average length of helices in the protein [52]. We have to note that BeStSel defines helix as α-helix while the CONTIN definition includes α-helix and 3_10_-helix. Usually the 3_10_-helix content is low or absent and we expect similar results for the two algorithms.

## Results and Discussion

### Stl protein is mainly α-helical

**Fig 1:**
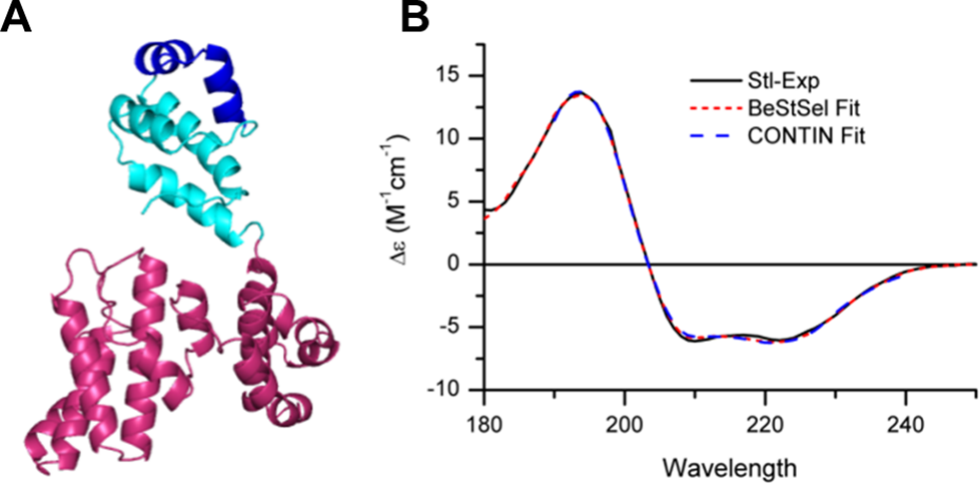
(A) Homology model of the Stl protein. Ribbon representation of the homology model of the *Staphylococcus aureus* pathogenicity island repressor Stl produced by Phyre2 Server [38]. Based on the homology model the protein is highly α helical (74%), and seems to be divided into two segments: the amino terminal segment colored cyan and the carboxy-terminal segment colored hotpink. According to Pfam and NCBI CDD the protein is predicted to contain a helix-turn-helix DNA binding motif. The position of the HTH predicted by NPS@ server is colored to dark blue [44]. **(B) SRCD spectrum of Stl and fitted curves by BeStSel and CONTIN algorithms.** Stl was measured at 2.1 mg/ml concentration in 50 mM Hepes, 200 mM NaCl, pH 7.5.

In lack of atomic resolution structural information, the structure of Stl was analyzed by *in silico* methods and synchrotron radiation circular dichroism (SRCD) spectroscopy. The predicted homology model as provided by the Phyre2 Server [38] applying intensive mode is shown on Fig 1A, while the templates used for modeling are listed in Table A in S1 File. The Stl sequence was entirely covered by alignment with the template sequences, with the exception of a very short part of the N-terminus (residues 1–7), which were modeled by *ab initio* methods. Apart from these first few residues the confidence of the alignment was more than 95%, so we assume that the overall fold and the core of the protein is modeled reliably, although the orientation of the surface loops are less well-defined. Based on this model, the repressor protein has a mostly α-helical (74%) secondary structure thus it is likely to belong to the class of “all-α” proteins (SCOP ID 46456) that includes various protein superfamilies. Related structures found by the Molecular Modeling Database (MMDB) with E-value > 10^−6^ (PDB ID: 2B5A, 3NTG, 2P5T) are also proteins with mostly α-helical fold [41]. Since the predicted fold type is shared by numerous proteins performing vast array of functions and the sequence identity between the templates and Stl are relatively low, we did not analyze the template proteins in detail. To compare this model with others generated by alternative structural annotation servers Stl sequence was submitted also to the HHpred predictor [39]. All templates used by Phyre2 (cf. Table A in S1 File) with the only exception of the human transcription factor Oct-1 were also within the list of closest homologs provided by HHpred. Following the HHpred search 3D structural models were generated by Modeller in two ways, i) with automatic template selection, which optimizes diversities of query and template HMMs, reranks templates and automatically selects best set, and ii) with the Phyre2 hits manually selected from the HHpred list as templates [40]. The first approach provided a well defined structural model only for the N-terminal residues (1–75), while the other resulted a structural model for almost all residues, however the C-terminal 33 residues are unstructured even in that model. Both the Phyre2 and the Modeller 3D structures agreed in that the protein is mostly α-helical and contains an N-terminal HTH motif (S1 Fig). However the orientation of the helices except the HTH was quite different in the two types of models.

Secondary structural elements of the full length Stl protein were also experimentally characterized by CD spectroscopy. CD spectroscopy is a frequently used method to assess protein conformation: tertiary structural elements can be well reflected in the near-UV wavelength range (250–380 nm) [53], while secondary structural elements show well-known spectral characteristic in the far-UV wavelength range (180–240 nm) [48,54]. Due to the high far-UV absorption of the 200 mM NaCl in the buffer, these measurements were performed using synchrotron radiation CD (SRCD) providing extended wavelength range and increased signal/noise ratio compared to conventional CD measurements. The observed spectral shape was characteristic of α-helical proteins (Fig 1B). The secondary structure content was quantitatively estimated from the CD spectrum by the BeStSel [47] and CONTIN [48] algorithms showing 67.6 and 63.3% α-helix content respectively (Table 1). For both algorithms, the predicted β-sheet content was low, being close to zero within the accuracy of the methods. Only the Phyre2 model was suitable for a comparison to the results of the SRCD measurements since it provided a 3D model for the whole protein (Table 1). The Stl Phyre-predicted 3D model shows similar secondary structural composition to that of the CD analysis within the experimental error supporting the reliability of the model. The two CD analysis methods also provided the possibility of estimation of the length and number of helical segments in the protein, which are in good agreement with the model, as well (Table 1). The accordance of the data obtained from the CD spectra and from the Phyre2 model, led us to consider this 3D model as a useful starting point for construct design and analysis tool for the Stl-like proteins.

**Table 1:** Secondary structure estimation from the synchrotron radiation CD spectrum of Stl and comparison to the homology model.

	BeStSel		CONTIN	
	CD analysis^a^	Model^b^	CD analysis^a^	Model^b^
Helix1	44.6	40.0	44.5	42.5
Helix2	23.0	21.4	18.8	21.4
β-sheet	3.6	0.0	6.3	0.0
Turn	8.4	7.5	9.0	10.4
Others	20.5	31.1	21.4	25.7
Number of helices	16	15	13	15
Average helix length	11.8	11.5	13.5	11.9

^a^The secondary structure composition from the CD spectrum was estimated by the BeStSel and CONTIN algorithms. The two algorithms use different secondary structure components, however, the overall helix, β-sheet and turn+others contents are comparable.

^b^The secondary structure contents were also calculated for the Phyre2 homology model using the DSSP algorithm [46] and the BeStSel and CONTIN definitions [47,48].

### Stl may possess two segments with distinct functions

The 3D homology model revealed that the Stl protein– as numerous other repressors –seemingly consists of two segments, which may fold independently (Fig 1A). Pfam and NCBI CDD protein domain annotation engines [42,43] predicted that Stl contains a helix-turn-helix DNA binding motif (HTH) at the amino-terminal part of the protein (between residues 15–68; with expectance value of 7.97 ·10^−11^). The HTH was predicted to reside between residues 27–48 with 100% probability by NPS@ server [44]. In addition, we used the MetaDisorder server [45] to estimate flexibility characteristics of the Stl protein (S2 Fig). Although these flexibility predictors provided rather different pattern, most agreed in indicating that a somewhat more ordered segment residues between residues 95–150, C-terminal to the HTH motif.

Based on these *in silico* predictions we hypothesized that the Stl, similarly to other well-known repressors, possesses two segments, with putative distinct functions. We hypothesize that the amino-terminal segment encodes DNA binding function, while the carboxy-terminal segment may be responsible for other protein-protein interactions. In the context of the Stl protein, complex formation with another protein partner, namely phage dUTPase was already shown to exist [10]. Hence, we speculated that this C-terminal segment may be involved in binding to phage dUTPase. We set out to investigate these suggestions and asked whether the N-terminal and C-terminal segments may fold independently and may still provide either DNA-binding or dUTPase-interacting function. To define the boundaries of the designed constructs, we relied on the i) the results from the flexibility annotation server (S2 Fig) ii) the Phyre2 structural prediction iii) length of the N-terminal protein constructs in experimentally determined three-dimensional structures of DNA-bound bacteriophage repressors [55–60]. With these considerations, a truncated construct encoding the C-terminal segment (residues 84–263) was produced by PCR based cloning and a stop codon was mutated to the full length sequence to obtain a construct for the N-terminal segment of Stl (residues 1–84). The expression of N-terminal segment proved to be unsuccessful even if attaching this segment to a GST-tag, indicating that the N-terminus of Stl, truncated at residue 85, may not fold independently. It was therefore not straightforward to analyze the N-terminal segment on its own. Hence we later took the approach to mutate the key residues in the HTH motif within the full-length context. The expression of the C-terminal segment was successful, indicating that this segment might be considered as an independently folded C-terminal domain (Stl-CTD).

### The Stl-CTD domain is not capable of DNA-binding but binds and inhibits dUTPase

**Fig 2:**
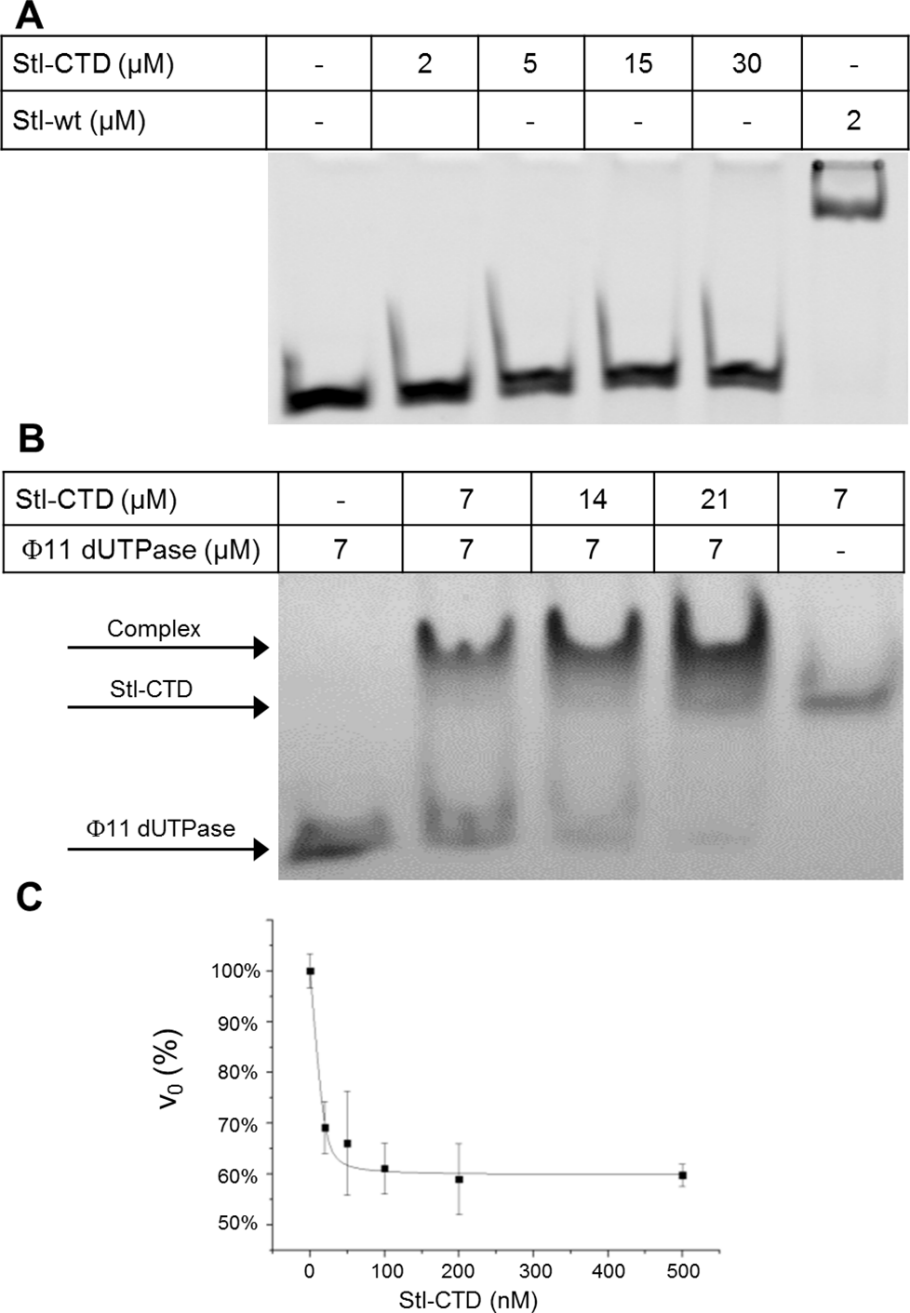
DNA and protein binding ability of Stl-CTD. **(A) Electrophoretic mobility shift assay** was performed to investigate the DNA binding ability of Stl-CTD. Species and concentrations given in monomers are indicated on the figure. The band of the dsDNA is only shifted upwards if wild type Stl is added but there is no shift even at high concentrations Stl-CTD. **(B) Native gel electrophoresis** experiment was performed to investigate the dUTPase binding ability of Stl-CTD. Species and concentrations given in monomers are indicated. The mixture of Stl-CTD and Φ11 dUTPase shows up in distinct position comparing to the individual proteins, which clearly indicate the complex formation. **(C) Activity of the Φ11 dUTPase** was measured in the presence and absence of Stl-CTD. Each measurement was repeated three times. The quadratic binding equation was fit to the data resulted in the apparent K_i_ = 1.5 ± 0.5 nM. The total change in amplitude of the activity was 40%.

**Fig 3:**
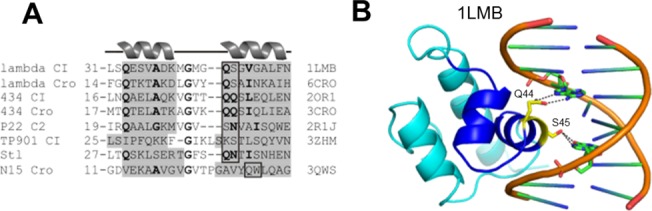
DNA binding domain of bacteriophage repressors. **(A) Sequence alignment of the HTH motifs of bacteriophage repressors and Stl.** The number before each segment is the amino acid sequence number of the first residue in the sequence. Helices are with gray background, similar residues are in bold, box highlights residues interacting with DNA nucleobases. PDB ID of the proteins is indicated on the right side of the sequences. **(B) Experimentally determined structure of the DNA-bound CI bacteriophage repressor** DNA cartoons orange, protein cartoon: dark blue for HTHs, otherwise cyan. DNA bases and DNA interacting amino acid residues are stick representation with atomic coloring (protein carbon yellow, DNA carbon green, oxygen red, nitrogen blue, phosphorus orange.) The PDB ID of the structure is indicated. Stereo representation of all experimentally determined protein-DNA complex structures represented in the sequence alignment in this figure are available in S3–S9 Figs.

To check the suggested domain functions, the DNA binding ability of Stl-CTD was investigated with electrophoretic mobility shift assay. Increasing concentrations (from 2 μM up to 30 μM) of Stl-CTD was added to 100 ng DNA and mixtures were run on native PAGE gel (Fig 2A). No DNA shift was observed in any of the Stl-CTD containing samples, while the positive control containing 2 μM Stl clearly showed the expected shift due to complex formation between Stl and DNA (cf Fig 3 in the present work and also Fig 2D in [10]).

The interaction of Stl-CTD with Φ11 dUTPase was tested by two independent methods. Native polyacrylamide gel electrophoresis experiment was performed with the mixture of the two proteins (Fig 2B). Similarly to our previous results with wild type full length Stl [10], the band corresponding to the complex of Stl-CTD: Φ11 dUTPase shows up at a distinct position as compared to the positions of the two individual proteins. The interaction of the two proteins was also investigated by measuring the effect of Stl-CTD on dUTPase activity (Fig 2C). Stl-CTD inhibited the dUTPase activity with the apparent inhibitory constant of K_i_ = 1.5 ± 0.5 nM. However, in contrast to wild type full length Stl, which caused a practically complete loss of dUTPase activity if added in high excess [10], the maximum inhibitory effect exerted by Stl-CTD on dUTPase was about 40% even at saturating inhibitor concentration. The difference in maximal dUTPase inhibition as compared to the wild type Stl suggests that the amino-terminal segment of Stl may also contribute to the interaction between the two proteins. In summary, these data show that the C-terminal segment of Stl lacks the potential for DNA-binding while partially constitutes ability for dUTPase binding and inhibition. These observations suggest that while the C-terminal segment is capable folding on its own, it does not possess full functional capability for either of the two function of the full-length Stl protein.

### Specific point mutations within the putative helix-turn-helix motif of the Stl N-terminal segment result in decreased DNA binding ability

To verify the prediction for a helix-turn-helix (HTH) motif, point mutations were performed within the Stl protein to abolish DNA binding. Design of these mutations was based on experimentally determined three-dimensional structures of DNA-bound bacteriophage repressors. As of present, 3D structures for seven such proteins are available in the PDB, among these we have focused on the best resolution structures, for which the PDB IDs are as follow: 1LMB; 6CRO, 2OR1, 3CRO, 2R1J, 3ZHM, 3QWS [55–60]. Besides the structural similarity of these phage repressor HTHs, five of those showed high level of sequence similarity to each other (Fig 3A), forming the basis of HTH prediction [44]. These structures together with numerous additional studies have established that the residues responsible for specific DNA binding are situated on the second helix of the HTH [61–63]. As shown on Fig 3A, with the exception of N15 phage Cro repressor, two residues could be identified as being conserved in different repressor-related HTH motifs, and moreover, in the published 3D structures, the role of these residues were also well defined. Namely, the first two residues of the second helix of the HTH motif provide H-bonding interaction with DNA nucleobases (Fig 3, S3–S9 Figs). In the Stl protein, two similar polar residues show up at the same site within the predicted HTH motif (Fig 3A). In conclusion, based on the sequence alignment of the predicted Stl HTH with different HTHs and the superimposition of the Stl Phyre2 structural model on the crystal structures of these repressors, mutations of the polar residues Q40-N41 in the second helix were performed. To preserve the helical secondary structure while erasing the potential H-bonding ability, these two residues were exchanged into alanines. A similar double alanine mutational analysis was successfully performed with the TP901 repressor and yielded important insights [60].

**Fig 4:**
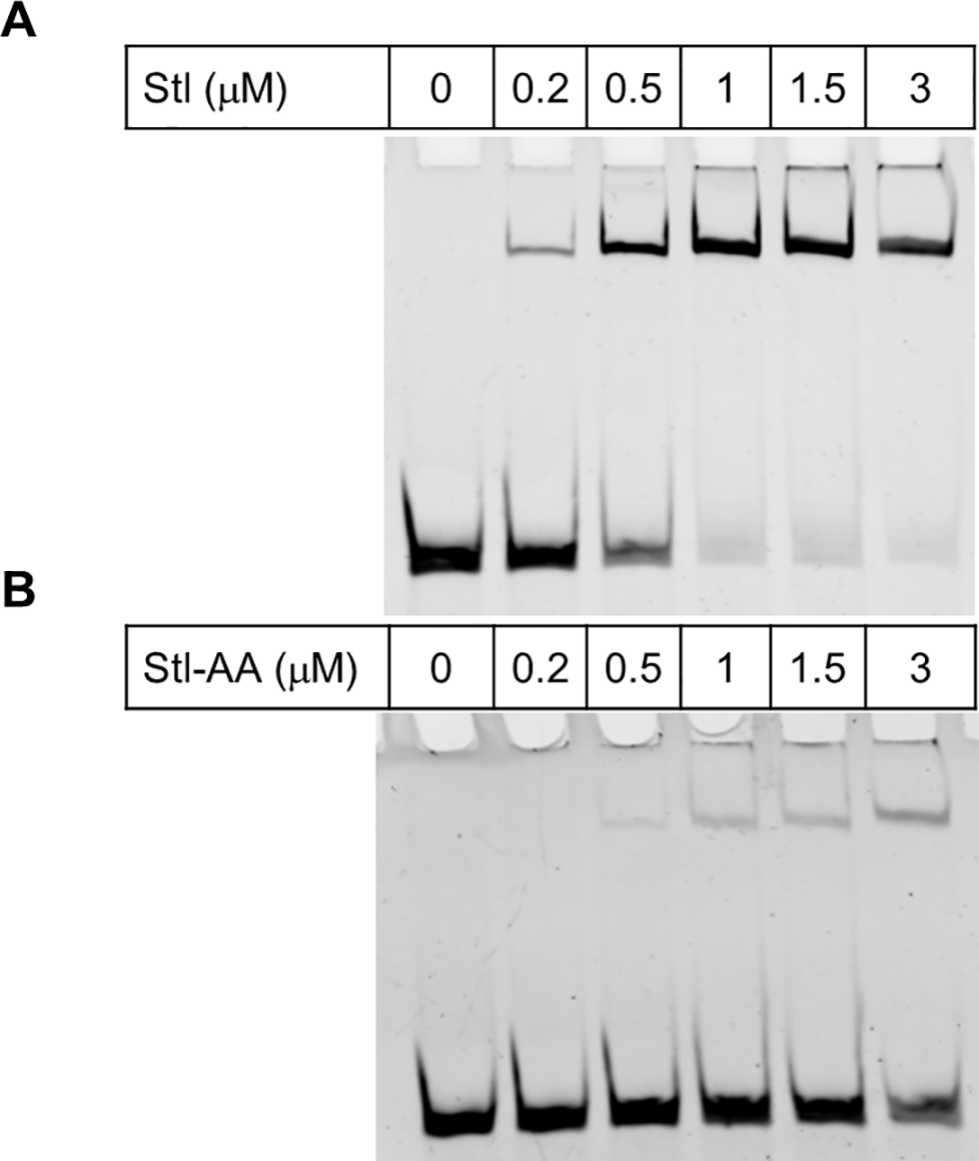
Electrophoretic mobility shift assay (EMSA) for testing the DNA binding ability of Stl constructs. **(A)** Result of EMSA with the wild type Stl protein. Note that the DNA band is shifted upwards at 1 μM Stl concentration. **(B)** EMSA gel result of Stl-AA mutant protein. Note that even at relatively high (ie. 3 μM) concentration of the Stl-AA mutant construct, most DNA still appears at the lower position on the gel, indicating lack of binding to the mutant protein.

According to the expectation, the double mutant (Q40-N41 – A40-A41) Stl construct (termed as Stl-AA) proved to be highly defective in DNA binding based on EMSA experiments (Fig 4, S10 Fig). To verify that the double mutations within the HTH motif did not perturb dUTPase binding ability, we checked whether the double mutant protein may still form a complex with dUTPase. We showed that the complex between Stl-AA and dUTPase is readily observable on native polyacrylamide gel electrophoresis (S11 Fig). Also, dUTPase inhibition by the Stl-AA mutant has practically the same characteristics as compared to the inhibitory effect of wild type Stl (S12 Fig): the inhibitory constant has been determined to be 1.4 ± 0.9 nM, and the maximal inhibition was above 90%. Comparing to the wild type Stl the inhibition effect of Stl-AA on Ф11 dUTPase is not perturbed [10]. Based on these findings, the design of an *in vivo* reporter system in in progress in our laboratory to test Stl-DNA and Stl-dUTPase interactions.

### General model for Stl-like repressors in *Staphylococci*

The group of Stl-like repressors has a major regulatory effect on the replication and subsequent horizontal transfer of mobile genetic elements in *Staphyloccoccal* strains. However, their structural and functional traits have not yet been addressed in details. In the present study, we have used the Phyre2 3D structural modeller software and the resulting model structure was in excellent agreement with experimentally determined structural elements via synchrotron radiation CD spectroscopy. Domain prediction based on this model did, in fact, make it possible to design an independently folding truncated construct (Stl-CTD). This domain lost DNA-binding capability but still preserved functionality with respect to binding and inhibition of dUTPase. Another *in silico* prediction method was also used with success in the present study: the HTH motif of Stl could be localized with confidence and this result could be fully ascertained by wetlab experiments (point mutations, EMSA and other assays).

**Fig 5:**
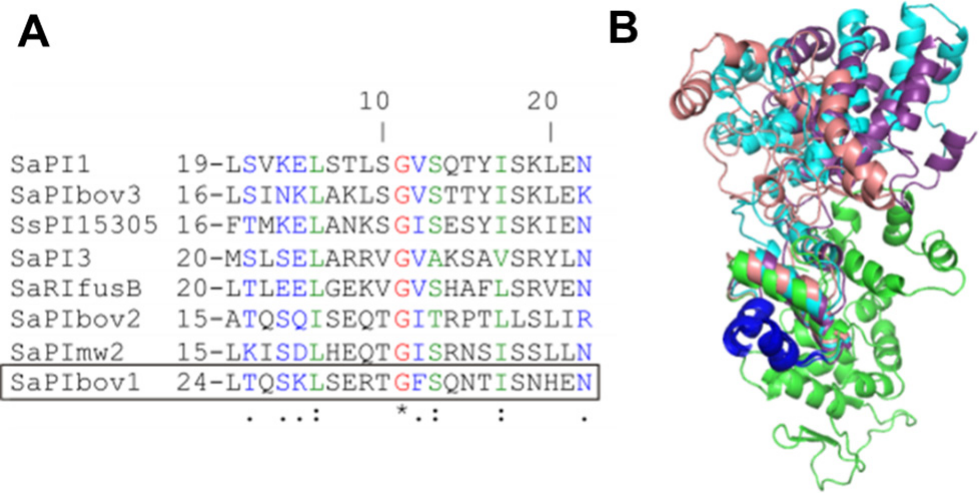
(A) Sequence alignment for the HTH segments of Stl-like repressors. Identical residues are in red (*), strongly similar (:) residues are in green, weakly similar residues are in blue (.). The protein investigated in the current study, termed as Stl throughput the text, is boxed (it is equivalent to the repressor of the SaPIbov1 pathogenicity island). **(B) Superimpositioned structural models of representative Stl-like repressors from different SaPIs.** Proteins are in cartoon representation. SaPIbov1 Stl is cyan, Stl-like repressors of SaPIbov3, SaPI1, and SsPI15305 pathogenicity islands are violetpurple, salmon, and green, respectively. Predicted HTH motifs of all proteins are colored dark blue. Stereo view of these structures is shown in S14 Fig.

Based on the success of these *in silico* predictions in the context of our Stl-focused study, we made an additional more generalized approach to decide if similar structural/functional elements may be identified in other representatives of the family of Stl-like repressors, as well. Towards this end, we first ran the HTH predictor on the different repressor protein sequences within the diverse *S*. *aureus* pathogenicity islands. Fig 5 shows an alignment for the HTH motif identified in the diverse repressors: out of the 12 repressor sequences, HTH could be identified in 8 proteins with a probability above 50% (Table B in S1 File). In all of these cases, the HTH motif is located at the N-terminal part of the proteins.

It is worthwhile to point out that there is considerable sequence conservation within these HTH segments, while the other parts of these repressor proteins show high diversity (cf. Fig 5 and S13 Fig). Protein Blast analysis of the Stl sequences did not find similarity among these proteins [64], however all of these were annotated as functioning as master repressors [65]. We have also used the Phyre2 modeler to build 3D models of these Stl-like repressors, to decide if these may possess similar 3D structures despite the high sequence diversity. Although these models are all characterized by a high α-helical content, i.e. similar folding pattern, the overall structures are still greatly diverse except for the well-conserved HTH motif (see structural models in Fig 5). It is rather probable that the HTH motifs are responsible for sequence-specific DNA binding in all of these repressors. For the case of the Sapibov1 Stl repressor, we have obtained detailed information of the mechanism of de-repression regulated by the dUTPase interacting partner [10]. Based on these data and the similar overall folding pattern, we propose that the carboxy-terminal domains of the other Stl-like repressors may constitute binding surface for interaction with other, yet unidentified proteins.

## Supporting Information

S1 File Document including Table A and Table B. (DOCX)

S1 Fig Comparison of the 3D models generated by Phyre2 and Modeller. **(A)** Ribbon representation of the homology model of the *Staphylococcus aureus* pathogenicity island repressor Stl produced by Phyre2 Server [38]. Based on the homology model the protein is highly α helical (74%), and seems to be divided into two segments: the amino terminal segment colored cyan and the carboxy-terminal segment colored hotpink. According to Pfam and NCBI CDD the protein is predicted to contain a helix-turn-helix DNA binding motif. The position of the HTH predicted by NPS@ server is colored to dark blue [44]. **(B)** Ribbon representation of the homology model of Stl obtained by Modeller [40], predicted HTH colored yellow. **(C)** Superimposition of the two models. Both models agreed in that the protein is mostly α-helical and contains an N-terminal HTH motif.
(TIF)

S2 Fig Disorder prediction and construct design. Representative curves from the results of flexibility prediction by MetaDisorder server are shown [45]. The mid-panel indicates our designed constructs represented as horizontal bars. Bottom-panel shows the secondary structure elements along the sequence, helices from the helix-turn-helix motif are in blue other helices are in purple.
(TIF)

S3 Fig Experimentally determined structure of the DNA-bound lambda bacteriophage CI repressor (PDB ID 1LMB). DNA cartoons orange, protein cartoons: dark blue for HTHs, otherwise cyan. DNA bases and DNA interacting amino acid residues are stick representation with atomic coloring (protein carbon yellow, DNA carbon green, oxygen red, nitrogen blue, phosphorus orange.)
(TIF)

S4 Fig Experimentally determined structure of the DNA-bound lambda bacteriophage Cro repressor (PDB ID 6CRO). Coloring as in S3 Fig.
(TIF)

S5 Fig Experimentally determined structure of the DNA-bound 434 bacteriophage CI repressor (PDB ID 2OR1). Coloring as in S3 Fig.
(TIF)

S6 Fig Experimentally determined structure of the DNA-bound 434 bacteriophage Cro repressor (PDB ID 3CRO). Coloring as in S3 Fig.
(TIF)

S7 Fig Experimentally determined structure of the DNA-bound P22 bacteriophage C2 repressor (PDB ID 2R1J). Coloring as in S3 Fig.
(TIF)

S8 Fig Experimentally determined structure of the DNA-bound TP901 bacteriophage CI repressor (PDB ID 3ZHM). Coloring as in S3 Fig.
(TIF)

S9 Fig Experimentally determined structure of the DNA-bound N15 bacteriophage Cro repressor (PDB ID 3QWS) Coloring as in S3 Fig. (TIF)

S10 Fig Testing the DNA binding ability of Stl-AA. Electrophoretic mobility shift assay was performed to investigate the DNA binding ability of Stl-AA. Species and concentrations given in monomers are indicated on the figure. The band of the dsDNA is only partially sifted upwards even if high concentrations of Stl-AA. Wild type Stl shows shift of the same amount of DNA at concentration of 1μM (cf. Fig 3).
(TIF)

S11 Fig Testing the interaction of Stl-AA with Ф11 dUTPase on native-PAGE. Native gel electrophoresis experiment was performed to investigate the Ф11 dUTPase binding ability of Stl-AA. Species and concentrations given in monomers are indicated. Comparing to the wild type Stl the complex formation of Stl-AA with Ф11 dUTPase is not perturbed (cf. Fig 1B. in [10]).
(TIF)

S12 Fig Testing the inhibition of Stl-AA with Ф11 dUTPase. Enzyme activity Ф11dUTPase was measured in mixtures containing different concentrations of Stl-AA. The inhibitory constant has been determined to be 1.4 ± 0.9 nM, and the maximal inhibition was above 90%. Comparing to the wild type Stl the inhibition effect of Stl-AA on Ф11 dUTPase is not perturbed (cf. Fig 2A. in [10]).
(TIF)

S13 Fig Alignment of different SaPI Stl proteins. Identical residues are red (*). Strongly similar (:) residues are green, weakly similar residues are blue (.). Residues in the predicted HTH are underlined.
(TIF)

S14 Fig Stereo view of superimpositioned structural models of representative Stl-like repressors from different SaPIs. Proteins in cartoon representation. SaPIbov1 Stl is cyan, Stl-like repressors of SaPIbov3, SaPI1, and SsPI15305 pathogenicity islands are violetpurple, salmon, and green, respectively. Predicted HTH motifs of all proteins colored dark blue.
(TIF)

## References

[1] Lindsay JA, Ruzin A, Ross HF, Kurepina N, Novick RP. The gene for toxic shock toxin is carried by a family of mobile pathogenicity islands in Staphylococcus aureus. Mol Microbiol. 1998;29: 527–543. 10.1046/j.1365-2958.1998.00947.x 9720870

[2] Ubeda C, Maiques E, Barry P, Matthews A, Tormo MA, Lasa I, et al SaPI mutations affecting replication and transfer and enabling autonomous replication in the absence of helper phage. Mol Microbiol. 2008;67: 493–503. 10.1111/j.1365-2958.2007.06027.x 18086210

[3] Tormo-Más MA, Mir I, Shrestha A, Tallent SM, Campoy S, Lasa I, et al Moonlighting bacteriophage proteins derepress staphylococcal pathogenicity islands. Nature. 2010;465: 779–82. 10.1038/nature09065 20473284 PMC3518041

[4] Vértessy BG, Tóth J. Keeping Uracil Out of DNA: Physiological Role, Structure and Catalytic Mechanism of dUTPases. Acc Chem Res. 2009;42: 97–106. 10.1021/ar800114w.Keeping 18837522 PMC2732909

[5] Persson R, Cedergren-Zeppezauer ES, Wilson KS. Homotrimeric dUTPases; structural solutions for specific recognition and hydrolysis of dUTP. Curr Protein Pept Sci. 2001;2: 287–300. 10.2174/1389203013381035 12369926

[6] Takács E, Grolmusz VK, Vértessy BG. A tradeoff between protein stability and conformational mobility in homotrimeric dUTPases. FEBS Lett. 2004;566: 48–54. 10.1016/j.febslet.2004.04.039 15147867

[7] Muha V, Horváth A, Békési A, Pukáncsik M, Hodoscsek B, Merényi G, et al Uracil-Containing DNA in Drosophila: Stability, Stage-Specific Accumulation, and Developmental Involvement. Hawley RS, editor. PLoS Genet. 2012;8: e1002738 10.1371/journal.pgen.1002738 22685418 PMC3369950

[8] Vertessy BG, Zalud P, Nyman PO, Zeppezauer M. Identification of tyrosine as a functional residue in the active site of Escherichia coli dUTPase. Biochim Biophys Acta. 1994;1205: 146–150. 10.1016/0167-4838(94)90103-1 8142479

[9] Hemsworth GR, González-Pacanowska D, Wilson KS. On the catalytic mechanism of dimeric dUTPases. Biochem J. 2013;456: 81–8. 10.1042/BJ20130796 24001052

[10] Szabó JE, Németh V, Papp-Kádár V, Nyíri K, Leveles I, Bendes AÁ, et al Highly potent dUTPase inhibition by a bacterial repressor protein reveals a novel mechanism for gene expression control. Nucleic Acids Res. 2014;42: 11912–20. 10.1093/nar/gku882 25274731 PMC4231751

[11] Hirmondó R, Szabó JE, Nyíri K, Tarjányi S, Dobrotka P, Tóth J, et al Cross-species inhibition of dUTPase via the Staphylococcal Stl protein perturbs dNTP pool and colony formation in Mycobacterium. DNA Repair (Amst). Elsevier B.V.; 2015;30: 21–27. 10.1016/j.dnarep.2015.03.005 25841100

[12] Varga B, Barabás O, Takács E, Nagy N, Nagy P, Vértessy BG. Active site of mycobacterial dUTPase: structural characteristics and a built-in sensor. Biochem Biophys Res Commun. 2008;373: 8–13. 10.1016/j.bbrc.2008.05.130 18519027

[13] Pecsi I, Hirmondo R, Brown AC, Lopata A, Parish T, Vertessy BG, et al The dUTPase enzyme is essential in Mycobacterium smegmatis. PLoS One. 2012;7: e37461 10.1371/journal.pone.0037461 22655049 PMC3360063

[14] Horváti K, Bacsa B, Szabó N, Fodor K, Balka G, Rusvai M, et al Antimycobacterial activity of peptide conjugate of pyridopyrimidine derivative against Mycobacterium tuberculosis in a series of in vitro and in vivo models. Tuberculosis. 2015;95: 207–211. 10.1016/j.tube.2015.02.026 25728610

[15] Cedergren-Zeppezauer ES, Larsson G, Nyman PO, Dauter Z, Wilson KS. Crystal structure of a dUTPase. Nature. Nature Publishing Group; 1992;355: 740–3. 10.1038/355740a0 1311056

[16] Larsson G, Nyman PO, Kvassman JO. Kinetic characterization of dUTPase from Escherichia coli. J Biol Chem. 1996;271: 24010–24016. 10.1074/jbc.271.39.24010 8798636

[17] Barabás O, Pongrácz V, Kovári J, Wilmanns M, Vértessy BG. Structural insights into the catalytic mechanism of phosphate ester hydrolysis by dUTPase. J Biol Chem. 2004;279: 42907–15. 10.1074/jbc.M406135200 15208312

[18] Takács E, Barabás O, Petoukhov M V, Svergun DI, Vértessy BG. Molecular shape and prominent role of beta-strand swapping in organization of dUTPase oligomers. FEBS Lett. 2009;583: 865–71. 10.1016/j.febslet.2009.02.011 19302784

[19] Pecsi I, Leveles I, Harmat V, Vertessy BG, Toth J. Aromatic stacking between nucleobase and enzyme promotes phosphate ester hydrolysis in dUTPase. 2010;38: 7179–7186. 10.1093/nar/gkq584 20601405 PMC2978360

[20] Leveles I, Németh V, Szabó JE, Harmat V, Nyíri K, Bendes ÁÁ, et al Structure and enzymatic mechanism of a moonlighting dUTPase. Acta Crystallogr Sect D Biol Crystallogr. International Union of Crystallography; 2013;69: 2298–2308. 10.1107/S0907444913021136 24311572

[21] Nagy GN, Leveles I, Vértessy BG. Preventive DNA repair by sanitizing the cellular (deoxy)nucleoside triphosphate pool. FEBS J. 2014;281: 4207–23. 10.1111/febs.12941 25052017

[22] Tormo-Más MÁ, Mir-Sanchis I, Shrestha A, Tallent SM, Campoy S, Lasa Í, et al Moonlighting bacteriophage proteins derepress staphylococcal pathogenicity islands. Nature. 2010;465: 779–82. 10.1038/nature09065 20473284 PMC3518041

[23] Mir-Sanchis I, Martínez-Rubio R, Martí M, Chen J, Lasa Í, Novick RP, et al Control of Staphylococcus aureus pathogenicity island excision. Mol Microbiol. 2012;85: 833–45. 10.1111/j.1365-2958.2012.08145.x 22742067

[24] Stayrook S, Jaru-Ampornpan P, Ni J, Hochschild A, Lewis M. Crystal structure of the lambda repressor and a model for pairwise cooperative operator binding. Nature. 2008;452: 1022–5. 10.1038/nature06831 18432246

[25] Hochschild A, Lewis M. The bacteriophage lambda CI protein finds an asymmetric solution. Current Opinion in Structural Biology. 2009 pp. 79–86. 10.1016/j.sbi.2008.12.008 19181516 PMC2684985

[26] Pinkett HW, Shearwin KE, Stayrook S, Dodd IB, Burr T, Hochschild A, et al The structural basis of cooperative regulation at an alternate genetic switch. Mol Cell. 2006;21: 605–15. 10.1016/j.molcel.2006.01.019 16507359

[27] Carlson PA, Koudelka GB. Expression, purification, and functional characterization of the carboxyl-terminal domain fragment of bacteriophage 434 repressor. J Bacteriol. 1994;176: 6907–6914. 7961451 10.1128/jb.176.22.6907-6914.1994 PMC197060

[28] Donner AL, Carlson PA, Koudelka GB. Dimerization specificity of P22 and 434 repressors is determined by multiple polypeptide segments. J Bacteriol. 1997;179: 1253–1261. 9023209 10.1128/jb.179.4.1253-1261.1997 PMC178823

[29] Révet B, Von Wilcken-Bergmann B, Bessert H, Barker A, Müller-Hill B. Four dimers of λ repressor bound to two suitably spaced pairs of λ operators form octamers and DNA loops over large distances. Curr Biol. 1999;9: 151–154. 10.1016/S0960-9822(99)80069-4 10021390

[30] Bell CE, Lewis M. Crystal Structure of the cl Repressor C-terminal Domain Octamer. J Mol Biol. 2001;314: 1127–1136. 10.1006/jmbi.2001.5196 11743728

[31] Roberts JW, Roberts CW. Proteolytic cleavage of bacteriophage lambda repressor in induction. Proc Natl Acad Sci U S A. 1975;72: 147–151. 10.1073/pnas.72.1.147 1090931 PMC432259

[32] Shearwin KE, Brumby AM, Egan JB. The tum protein of coliphage 186 is an antirepressor. J Biol Chem. 1998;273: 5708–5715. 10.1074/jbc.273.10.5708 9488703

[33] Heinzel T, Velleman M, Schuster H. C1 Repressor of phage P1 is inactivated by noncovalent binding of P1 coi protein. J Biol Chem. 1992;267: 4183–4188. 1740459

[34] Heinzel T, Velleman M, Schuster H. The c1 repressor inactivator protein coi of bacteriophage P1: Cloning and expression of coi and its interference with c1 repressor function. J Biol Chem. 1990;265: 17928–17934. 2211669

[35] Riedel HD, Heinrich J, Heisig A, Choli T, Schuster H. The antirepressor of phage P1. Isolation and interaction with the C1 repressor of P1 and P7. FEBS Lett. 1993;334: 165–169. 10.1016/0014-5793(93)81705-5 8224242

[36] Rohs R, Jin X, West SM, Joshi R, Honig B, Mann RS. Origins of specificity in protein-DNA recognition. Annu Rev Biochem. 2010;79: 233–269. 10.1146/annurev-biochem-060408-091030 20334529 PMC3285485

[37] Wojciak JM, Iwahara J, Clubb RT. The Mu repressor-DNA complex contains an immobilized “wing” within the minor groove. Nat Struct Biol. 2001;8: 84–90. 10.1038/83103 11135677

[38] Kelley L a, Sternberg MJE. Protein structure prediction on the Web: a case study using the Phyre server. Nat Protoc. 2009;4: 363–71. 10.1038/nprot.2009.2 19247286

[39] Söding J. Protein homology detection by HMM-HMM comparison. Bioinformatics. 2005;21: 951–60. 10.1093/bioinformatics/bti125 15531603

[40] Eswar N, Webb B, Marti-Renom MA, Madhusudhan MS, Eramian D, Shen M-Y, et al Comparative protein structure modeling using Modeller. Current Protocols in Bioinformatics. John Wiley & Sons, Inc.; 2006 p. Unit 5.6. 10.1002/0471250953.bi0506s15 18428767 PMC4186674

[41] Wang Y, Addess KJ, Chen J, Geer LY, He J, He S, et al MMDB: annotating protein sequences with Entrez’s 3D-structure database. Nucleic Acids Res. 2007;35: D298–300. 10.1093/nar/gkl952 17135201 PMC1751549

[42] Finn RD, Bateman A, Clements J, Coggill P, Eberhardt RY, Eddy SR, et al Pfam: the protein families database. Nucleic Acids Res. 2014;42: D222–30. 10.1093/nar/gkt1223 24288371 PMC3965110

[43] Marchler-Bauer A, Derbyshire MK, Gonzales NR, Lu S, Chitsaz F, Geer LY, et al CDD: NCBI’s conserved domain database. Nucleic Acids Res. 2014;43: 222–226. 10.1093/nar/gku1221 PMC4383992 25414356

[44] Dodd IB, Egan JB. Improved detection of helix-turn-helix DNA-binding motifs in protein sequences. Nucleic Acids Res. 1990;18: 5019–5026. 10.1093/nar/18.17.5019 2402433 PMC332109

[45] Kozlowski LP, Bujnicki JM. MetaDisorder: a meta-server for the prediction of intrinsic disorder in proteins. BMC Bioinformatics. BMC Bioinformatics; 2012;13: 111 10.1186/1471-2105-13-111 22624656 PMC3465245

[46] Kabsch W, Sander C. Dictionary of protein secondary structure: pattern recognition of hydrogen-bonded and geometrical features. Biopolymers. 1983;22: 2577–2637. 10.1002/bip.360221211 6667333

[47] Micsonai A, Wien F, Kernya L, Lee Y-H, Goto Y, Réfrégiers M, et al Accurate secondary structure prediction and fold recognition for circular dichroism spectroscopy. Proc Natl Acad Sci U S A. 2015;112: E3095–E3103. 10.1073/pnas.1500851112 26038575 PMC4475991

[48] Provencher SW, Glöckner J. Estimation of globular protein secondary structure from circular dichroism. Biochemistry. 1981;20: 33–37. 10.1021/bi00504a006 7470476

[49] Leveles I, Róna G, Zagyva I, Bendes Á, Harmat V, Vértessy BG. Crystallization and preliminary crystallographic analysis of dUTPase from the φ11 helper phage of Staphylococcus aureus. Acta Crystallogr Sect F. 2011;67: 1411–3. 10.1107/S1744309111034580 PMC3212463 22102244

[50] Vertessy BG. Flexible glycine rich motif of Escherichia coli deoxyuridine triphosphate nucleotidohydrolase is important for functional but not for structural integrity of the enzyme. Proteins Struct Funct Genet. 1997;28: 568–579. 10.1002/(SICI)1097-0134(199708)28:4<568::AID-PROT10>3.0.CO;2-E 9261872

[51] Chen GC, Yang JT. Two-Point Calibration of Circular Dichrometer with d-10-Camphorsulfonic Acid. Anal Lett. Taylor & Francis Group; 1977;10: 1195–1207.

[52] Sreerama N, Venyaminov SY, Woody RW. Estimation of the number of alpha-helical and beta-strand segments in proteins using circular dichroism spectroscopy. Protein Sci. 1999;8: 370–80. 10.1110/ps.8.2.370 10048330 PMC2144265

[53] Orosz F, Kovács J, Löw P, Vértessy BG, Urbányi Z, Acs T, et al Interaction of a new bis-indol derivative, KAR-2 with tubulin and its antimitotic activity. Br J Pharmacol. 1997;121: 947–954. 10.1038/sj.bjp.0701189 9222552 PMC1564756

[54] Whitmore L, Wallace BA. Protein secondary structure analyses from circular dichroism spectroscopy: methods and reference databases. Biopolymers. 2008;89: 392–400. 10.1002/bip.20853 17896349

[55] Beamer LJ, Pabo CO. Refined 1.8 A crystal structure of the lambda repressor-operator complex. J Mol Biol. 1992;227: 177–196. 10.1016/0022-2836(92)90690-L 1387915

[56] Albright RA, Matthews BW. Crystal structure of lambda-Cro bound to a consensus operator at 3.0 A resolution. J Mol Biol. 1998;280: 137–151. 10.1006/jmbi.1998.1848 9653037

[57] Aggarwal AK, Rodgers DW, Drottar M, Ptashne M, Harrison SC. Recognition of a DNA operator by the repressor of phage 434: a view at high resolution. Science. 1988;242: 899–907. 10.1126/science.3187531 3187531

[58] Shimon LJ, Harrison SC. The phage 434 OR2/R1-69 complex at 2.5 A resolution. J Mol Biol. 1993;232: 826–838. 10.1016/0022-2836(91)90568-Q 8355273

[59] Watkins D, Hsiao C, Woods KK, Koudelka GB, Williams LD. P22 c2 repressor-operator complex: Mechanisms of direct and indirect readout. Biochemistry. 2008;47: 2325–2338. 10.1021/bi701826f 18237194

[60] Pedersen M, Lo Leggio L, Grossmann JG, Larsen S, Hammer K. Identification of quaternary structure and functional domains of the CI repressor from bacteriophage TP901-1. J Mol Biol. Elsevier Ltd; 2008;376: 983–96. 10.1016/j.jmb.2007.12.022 18191944

[61] Isackson PJ, Bertrand KP. Dominant negative mutations in the Tn10 tet repressor: evidence for use of the conserved helix-turn-helix motif in DNA binding. Proc Natl Acad Sci U S A. 1985;82: 6226–6230. 10.1073/pnas.82.18.6226 2994067 PMC391025

[62] Brennan RG, Matthews BW. The helix-turn-helix DNA binding motif. J Biol Chem. 1989;264: 1903–1906. 2644244

[63] Aravind L, Anantharaman V, Balaji S, Babu MM, Iyer LM. The many faces of the helix-turn-helix domain: transcription regulation and beyond. FEMS Microbiol Rev. 2005;29: 231–62. 10.1016/j.femsre.2004.12.008 15808743

[64] Johnson M, Zaretskaya I, Raytselis Y, Merezhuk Y, McGinnis S, Madden TL. NCBI BLAST: a better web interface. Nucleic Acids Res. 2008;36: W5–9. 10.1093/nar/gkn201 18440982 PMC2447716

[65] Novick RP, Christie GE, Penadés JR. The phage-related chromosomal islands of Gram-positive bacteria. Nat Rev Microbiol. 2010;8: 541–51. 10.1038/nrmicro2393 20634809 PMC3522866

