# Supplementary material for: Evidence-Based Structural Model of the Staphylococcal Repressor Protein: Separation of Functions into Different Domains
Source: PLoS One. 2015 Sep 28;10(9):e0139086. doi: 10.1371/journal.pone.0139086 (PMC4634304; doi:10.1371/journal.pone.0139086)
Supplement: S1 File — (DOCX) [file pone.0139086.s001.docx]

**Supporting Information**

**Evidence-based structural model of the Staphylococcal repressor protein: separation of functions into different domains**

Kinga Nyíri^1,2,*^, Bianka Kőhegyi^1,2^, András Micsonai^3^, József Kardos^3^, Beáta G. Vértessy^1,2,*^

^1^Department of Applied Biotechnology and Food Sciences, Budapest University of Technology and Economics, Budapest, 1111, Hungary, ^2^Institute of Enzymology, Research Centre for Natural Sciences, Hungarian Academy of Sciences, Budapest, 1117, Hungary and ^3^Department of Biochemistry and MTA-ELTE NAP B Neuroimmunology Research Group, Institute of Biology, Eötvös Loránd University, Budapest,1117, Hungary

* To whom correspondence should be addressed. Tel:+36 1 382 6707; Fax: +36 1 463 3855; Email: vertessy@mail.bme.hu, vertessy.beata@ttk.mta.hu
Correspondence may also be addressed to Kinga Nyíri. Tel:+36 1 463 1401; Fax: +36 1 463 3855; Email: [nyiri.kinga@ttk.mta.hu](mailto:nyiri.kinga@ttk.mta.hu)

**SUPPLEMENTARY TABLES**

**Table A Sequence coverage of Stl by templates and confidence values of the match to that template overall in the Phyre2 model** [1]**.**

| **Template**  **PDB code** | **Protein Name and Function** | **Aligned**  **residues*** | **Alignment Coverage* (%)** | **Confidence****  **(%)** | **Identity**  **(%)** |
| --- | --- | --- | --- | --- | --- |
| 1E3O | Human transcription factor Oct-1. activator of promoters of genes for some small nuclear RNAs (snRNA), histone H2B and immunoglobulins | 2-48 | 17^#^ | 96.5 | 33^#^ |
| 4YV9 | Rgg protein, transcriptional regulator of *Streptococcus dysgalactiae* | 14-263 | 93 | 99.7 | 12 |
| 2GRM | Prgx, molecular switch controlling expression of conjugation and virulence genes encoded by plasmid pCF10 of *Enterococcus faecalis* | 14-261 | 92 | 99.8 | 12^i^ |
| 4RYK | Lmo0325 protein, a putative transcriptional regulator from *Listeria* *monocytogenes* EGD-e | 13-260 | 92 | 99.8 | 12 |
| 2QFC | PlcR, major virulence regulator of the *Bacillus cereus* group | 12-261 | 93 | 99.7 | 13 |
| 2AXZ | Prgx, molecular switch controlling expression of conjugation and virulence genes encoded by plasmid pCF10 of *Enterococcus faecalis* | 14-261 | 92 | 99.7 | 13^i^ |
| 2EBY | ybaQ, putative HTH-type transcriptional regulator from *E. coli* | 11-96 | 31 | 99.6 | 15 |

* Part of Stl sequence which is covered by alignment with the template sequence.

** Confidence represents the estimated precision, it is the probability that the match between the query sequence and a template is because of true homology [1].

^#^ This template was included to the final model by the Phyre2 because of higher local similarity.

^i^ The difference in the sequence identity of Stl with the same type of protein occurs because the PDB entry 2GRM contains the structure of a longer protein construct and more residues are modeled in that structure, than those in 2AXZ, therefore the Phyre2 alignment differs somewhat in the two cases.

**Table B Sequences and NCBI Reference Sequence of different SaPI Stl proteins.**

| **SaPI Name*** | **NCBI Ref. Seq.** | **Sequence** (Predicted HTH in red) [2] |
| --- | --- | --- |
| **SaPI4** | EFG58984.1 | MVESNDELKKELGRFLKSIRKQKGKTASEISKQMQYSQGHISGIENGVKSFPSNKLIESYLMNIKDTNEEYNFYVDEIAKITKNKVKLNKVSNVTNKMGIIDRMMDIPYSREFISFDDNNEKSFTIFNISINDLHFHLQDINNYKFYKGIRLTDNDKNNIDKILNNYFENKSVIIKENTKTLRDKNENWEQLVKLSDYIDDKLDKKN |
| **SaPI1028** | YP_239447.1 | **97% identity with SaPI1** |
| **SaPIbov1** | AF217235.1 | MEGAGQMAELPTHYGTIIKTLRKYMKLTQSKLSERTGFSQNTISNHENGNRNIGVNGIEIYGKGLGIPSYILHRISDEFKEKGYSPTLNDFGKFDKMYSYVNKAYYNDGDIYYSSYDLYDETIKLLELLKESKINVNDIDYDYVLKLYKQILSTDTEKSIINYETLANTRKSSDKKREVTIEEIGEFHEKYLKLLFTNLETHNDRKKALAEIEKLKEESIYLGEKLRLVPNHHYDAIKGKPMYKLYLYEYPDRLEHQKKIILEKDTN |
| **SaPIbov2** | AAP55249.1 | MILCTLKNYMKLFGATQSQISEQTGITRPTLLSLIRNENKNIKYDTIDELCNFFGIQLKDLLIYSPVKIKQKSFNIKTIIEEYEHINESWKTYGVSIAYEINNEDFIFEGSIDPIDLKTFKNKKFENGTLYLNCNCFIEKDNYENLLKAGFSKEFFDLYNDLNQIKNKIVDKLPFELDSDLLIFNIFFNVRNAPSLEEYKEELQFLPTDSLVDLKNEIDKYLK |
| **SaPImw2** | WP_000583241.1 | MIIFRLKEIMEEKNLKISDLHEQTGISRNSISSLLNGKTRGIQFDTLEKITLALNVDVADLFKNVFNELIIKLDDISKVETYRRSKKFKEKKNIIVKKYAVNCDLIEDNDLKKGFIPYEISIELNPNPEIEIKIQFDYSNLFNYLIKFLEDCNNFKLLLVNYLSKKIYCLENKRINEIKSFYSIPDEKVYILSSFPGIFIRRPLRDNNGIFENIELNKIINGLNFNSNYNYTYSDQITLTHKNKK |
| **SaPIbov3** | WP_000672427.1 | MKFSEMLKKYRTKENLSINKLAKLSGVSTTYISKLEKNDRSYPTVEIIFNLAYGIIMKIKEKYDGIENSDDFLYPQIEEIISSFATSEDSNLDEENKNTIIDDFIMFMERKEKEFLNKSFGDNKEIYENKIALVSNSMNYKKTDYPYFDLKWLLSQNNFEVFYGRDFITNFATIEDSKLNTKSMYFYNILDKEDLKTIQRLIEVYLESKYPKIKDKDDFFVLATDKQNRIKNTIDWYNIN |
| **SaPIm4** | WP_000390814.1 | MEEFGEKFTHKAHKSIVSKWEKGLTKPSNERLKEIAKLGNISVHQLIYGDFLGLLESIANEEIKFILDTNMCANNSFLANELSSSVSRFIFSYYERGKENFNENLFRKLLQHYLQLELDLGNRDLESLTYFAYQRTINAQELVVDYYEDSKAKEFLKDESIDEFLTTISNKYFDLLEYIDDYRVKHDLEKISEE** |
| **ShPI2** | WP_011276365.1 | MYDKASIGKRIKEIRLKIGKTQTQFGDLFSASKGNVATWEKGVSLPNAKRLKEIARLGDMTTDQLLYGYNKDVYITIYNNLLENNPKETSVGKALRYPEKAVINELLKSAIEYTSEVPIFKDKIKSEEDILKNEFYYFIEKTFLDYYVKNHKSNNNILILAEDSITNIKSNISEYQFYDFNLQLPKIFNEATLLFDEYENSVSLELLKEIEKLSTNFLEEINKLKAKYPDNTPQRIINAQVFQNEPLKPLANYSFDIPDIKNNKDKQNYIFENLNTTINELIKDNPQLIKWINKNYIHKCDLNES |
| **SaPI1** | AAC28967.2 | MIYMTFGEILKKERVSWKLSVKELSTLSGVSQTYISKLENGKRNFPSLETIFNLLIGFKTHIEYKMGSESPFYEINNSYLDEILIMFINSSNSTISDRDPNELITQFNEYYDVTIKKKQNENSKIESDIFSNKIKLVKGTTKKEVIEKPYFDLNWLLTQNEYEVFFDRSFLLDNNFLNKKHFTEKDMYYYNVLNDNDLKTIKDLIVVFLLNKYNYIKNKDDFFNIFTNSEDDKTKRDALYKILYETD |
| **SaPI3** | WP_001260004.1 | MRTNDEIITIIKTSMKEQNMSLSELARRVGVAKSAVSRYLNLTREFPLNRAEDFAKVLGIKTEYLLGFAEREESTKQDTIAAHLDGDFTEEELIEIRKYAELVRKAHRNQ |
| **SaPI5** | ABD21675.1 | **Same as SaPI3.** |
| **SaPI2** | WP_000620857.1 | MIRNRLSELLSERGLKISRVAKDVKIARSSLTSMAQNDSEMIRYDAIDKLCSYLHISPSEFFEHNPINFDFTFDEEPNYKINDVFEGFEVTANITHAFSIENFDFEILVDVELDNRQKLNFDLDVSYKETEKITNSQHRFIFTIKNEDENIGLKKYVDSLSAGLKNLLFKKINQKLSGYVSEIIVKNIDDIEELFPNKGEKSTTLHKEILQTDSRLSSDIFKEY** |
| **SaPIm1/n1** | BAB43114.1 | **Same as SaPI2.** |
| **SaP122** | AJ938182.1 | **Same as SaPIbov1** |
| **SsPI15305** | BAE19091.1 | MSLNSLLKEQRKEKGFTMKELANKSGISESYISKIENNSVSLPKKERLLSLAYSLDPENKENLYSRFLTLASYNLENAEKEFNQFANMKQANLDKGLPNKKFTDNFVRIDKKNSKINSVEYPYFDLEWLLNQERFELFLGRTDVNILLDNNETEKELLILKDHERDRLRNIINIFKEGLLNERTKISREKDENVLISHSHEYTLIFDLLNKNIDDRNSLISQLGMINQNRDVFYEDNYYSAINQAVEQQDALKLQRLVRMTTINELKQYLSEQN |
| **SaRIfusB** | CAL23818.1 | MNQEGTLGHAIKSARKNYPLTLEELGEKVGVSHAFLSRVENNKITPNDKLLVKIANVLDFNETQDFLNEFRILAGYYDNIDENTAIFNNLKSSGRLEINRFKNEKKIVDKPYYKLNYLLESENKVFYDIKTSELGEKLVTIELPPGILHEIYKMINLEIIKTIKNNSKLLKSIENPQVIMEYQKEMEKTRKEFTQYLEKSLSTYDIDSVMSELYDDEYLI |

*According to [3]
** According to the prediction software, it is unlikely that this protein contains a HTH motif.

**Supplemental References**

1. Kelley L a, Sternberg MJE. Protein structure prediction on the Web: a case study using the Phyre server. Nat Protoc. 2009;4: 363–71. doi:10.1038/nprot.2009.2

2. Dodd IB, Egan JB. Improved detection of helix-turn-helix DNA-binding motifs in protein sequences. Nucleic Acids Res. 1990;18: 5019–5026. doi:10.1093/nar/18.17.5019

3. Novick RP, Christie GE, Penadés JR. The phage-related chromosomal islands of Gram-positive bacteria. Nat Rev Microbiol. 2010;8: 541–51. doi:10.1038/nrmicro2393
