# Supplementary figures and images for: Evidence-Based Structural Model of the Staphylococcal Repressor Protein: Separation of Functions into Different Domains
Source: PLoS One. 2015 Sep 28;10(9):e0139086. doi: 10.1371/journal.pone.0139086 (PMC4634304; doi:10.1371/journal.pone.0139086)

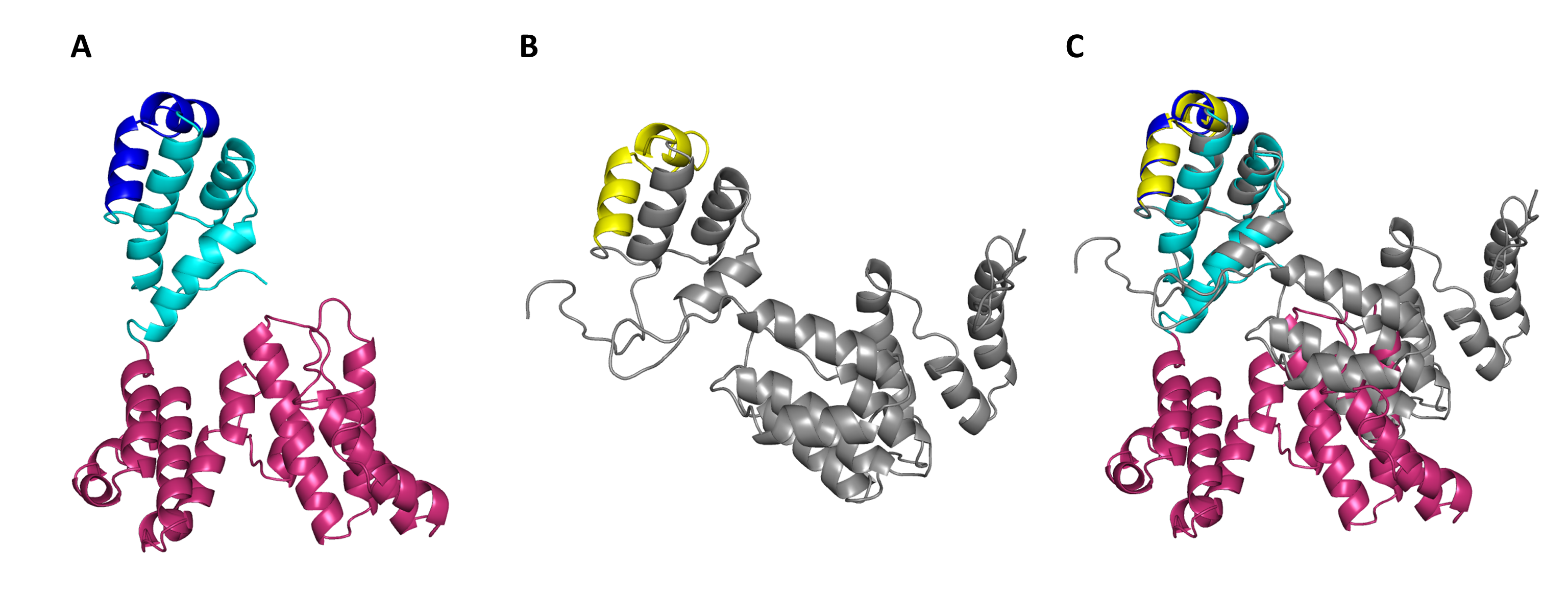

Supplement: S1 Fig — (A) Ribbon representation of the homology model of the Staphylococcus aureus pathogenicity island repressor Stl produced by Phyre2 Server [38]. Based on the homology model the protein is highly α helical (74%), and seems to be divided into two segments: the amino terminal segment colored cyan and the carboxy-terminal segment colored hotpink. According to Pfam and NCBI CDD the protein is predicted to contain a helix-turn-helix DNA binding motif. The position of the HTH predicted by NPS@ server is colored to dark blue [44]. (B) Ribbon representation of the homology model of Stl obtained by Modeller [40], predicted HTH colored yellow. (C) Superimposition of the two models. Both models agreed in that the protein is mostly α-helical and contains an N-terminal HTH motif. (TIF) [file pone.0139086.s002.tif]

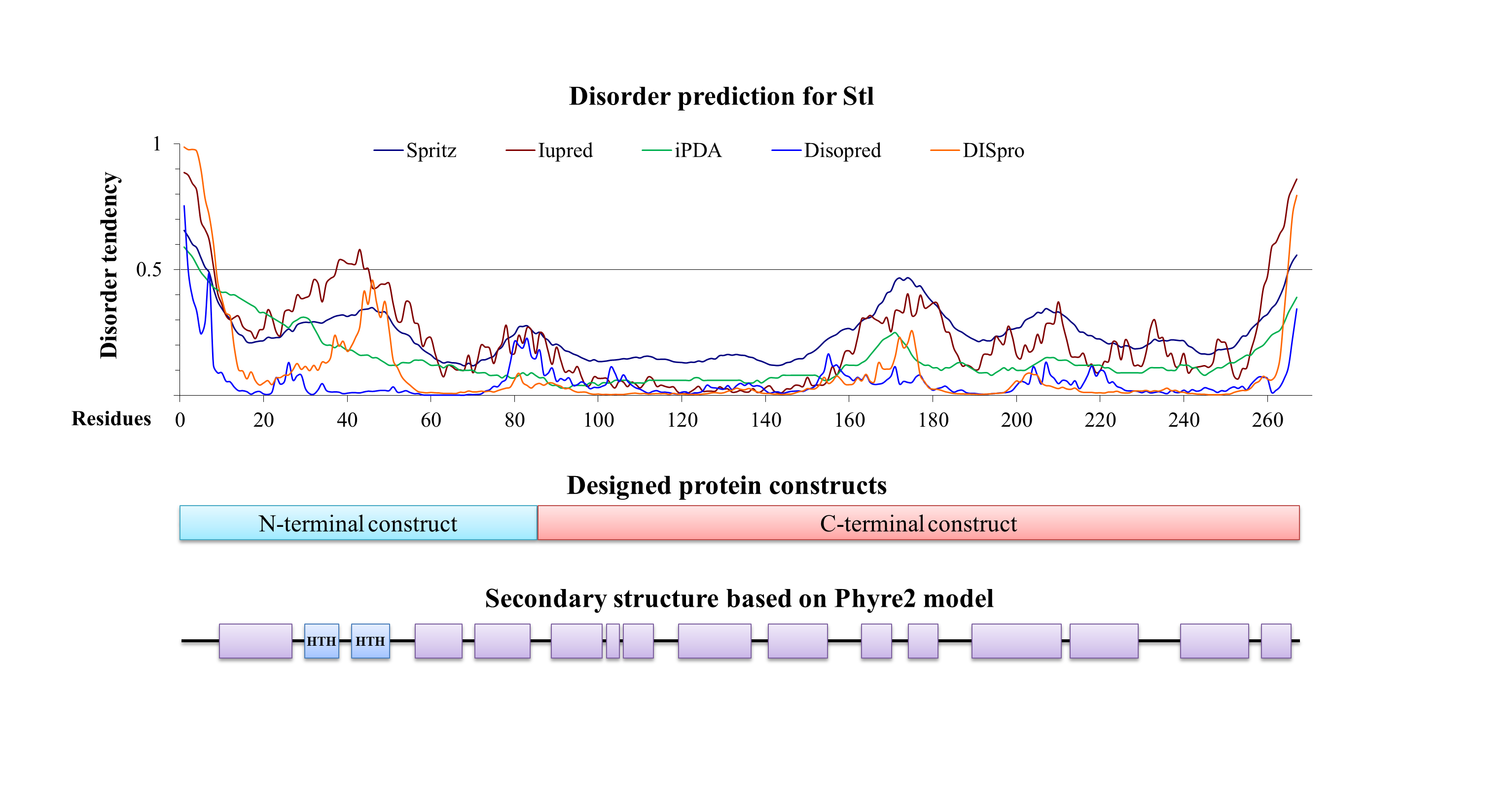

Supplement: S2 Fig — Representative curves from the results of flexibility prediction by MetaDisorder server are shown [45]. The mid-panel indicates our designed constructs represented as horizontal bars. Bottom-panel shows the secondary structure elements along the sequence, helices from the helix-turn-helix motif are in blue other helices are in purple. (TIF) [file pone.0139086.s003.tif]

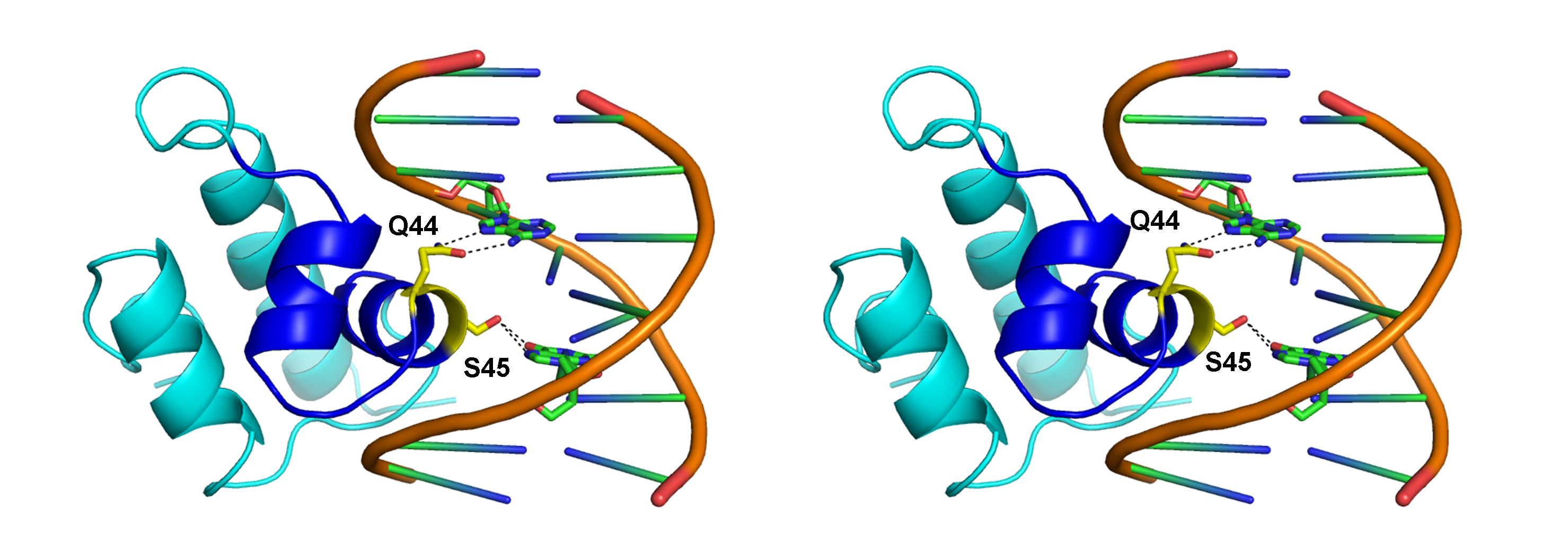

Supplement: S3 Fig — DNA cartoons orange, protein cartoons: dark blue for HTHs, otherwise cyan. DNA bases and DNA interacting amino acid residues are stick representation with atomic coloring (protein carbon yellow, DNA carbon green, oxygen red, nitrogen blue, phosphorus orange.) (TIF) [file pone.0139086.s004.tif]

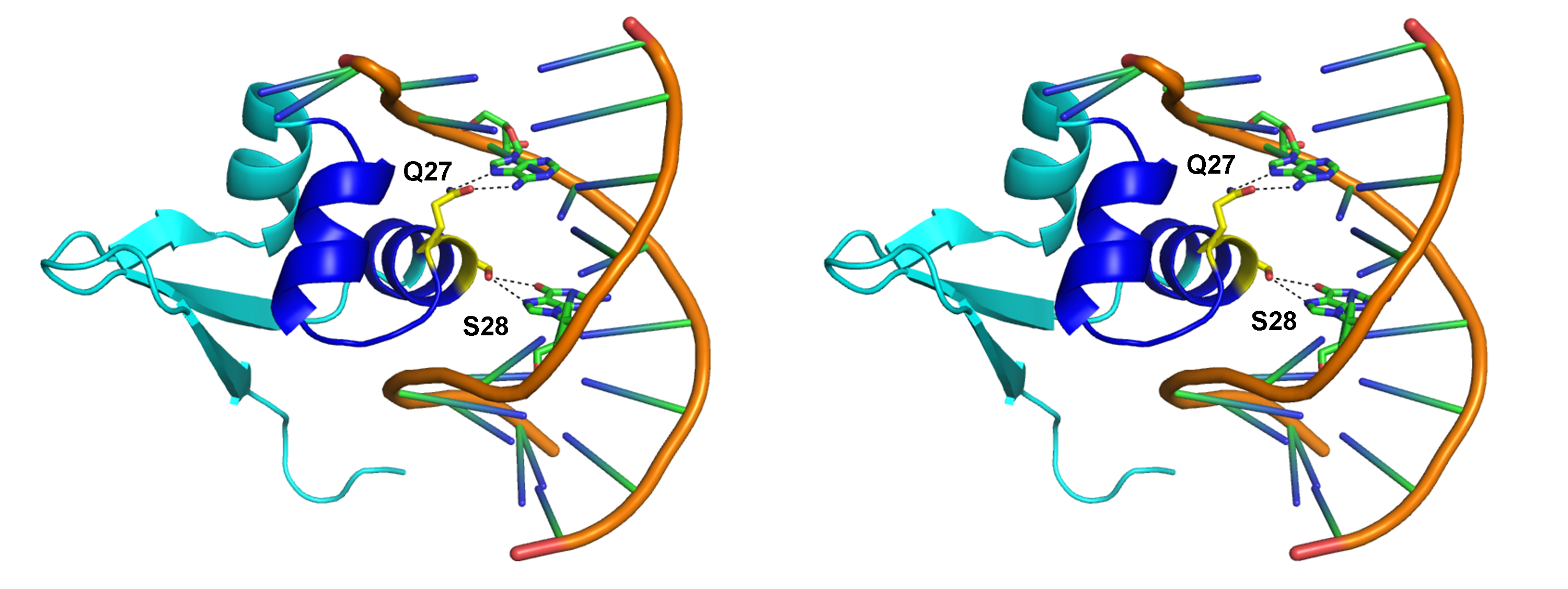

Supplement: S4 Fig — Coloring as in S3 Fig. (TIF) [file pone.0139086.s005.tif]

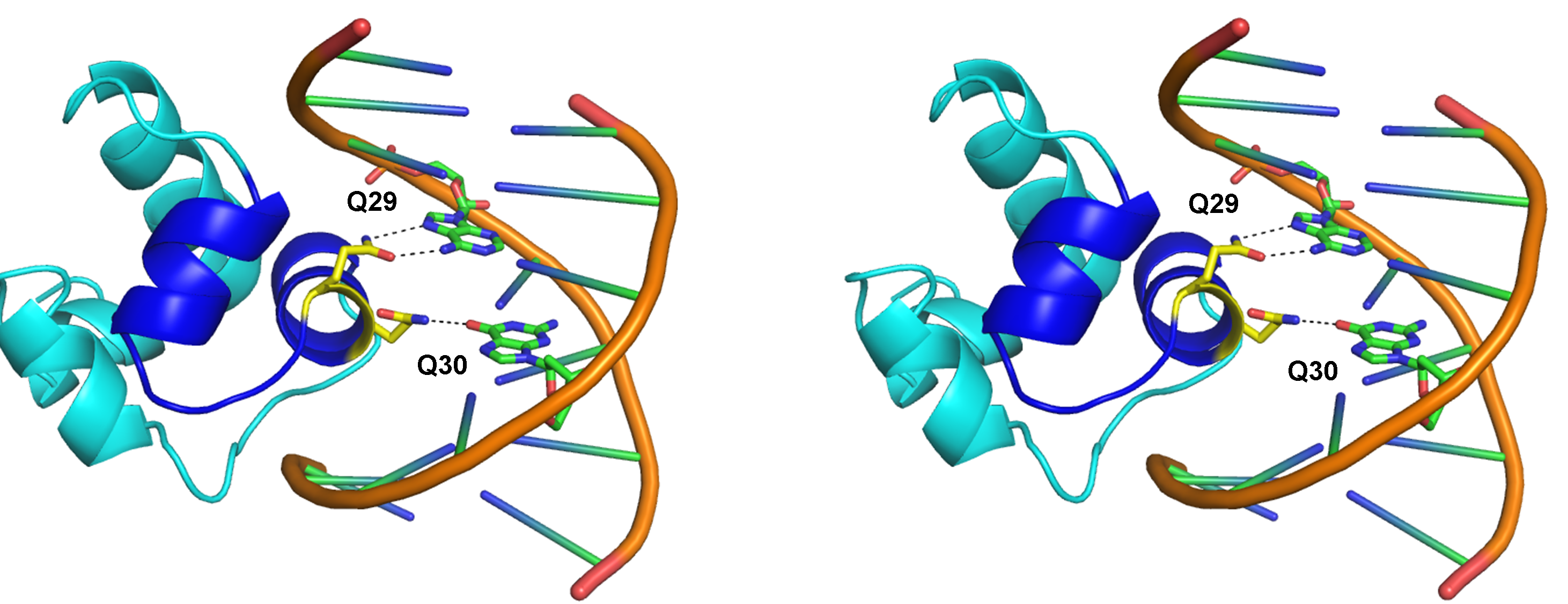

Supplement: S5 Fig — Coloring as in S3 Fig. (TIF) [file pone.0139086.s006.tif]

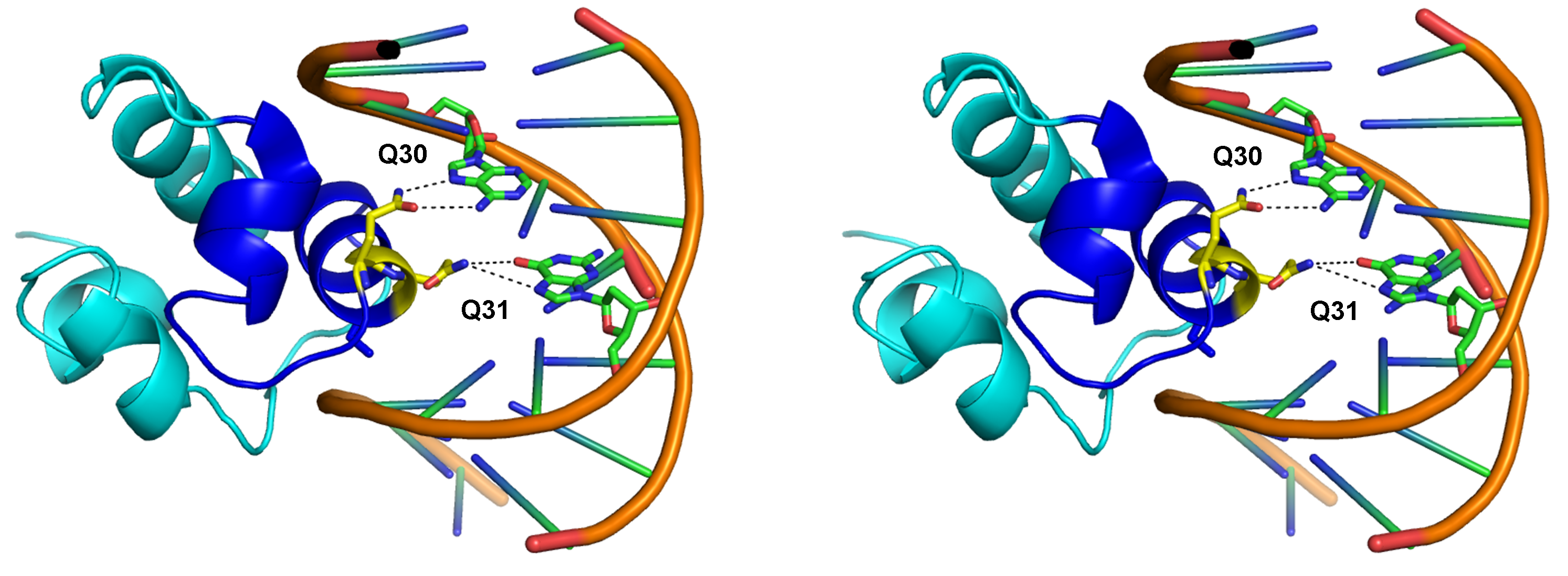

Supplement: S6 Fig — Coloring as in S3 Fig. (TIF) [file pone.0139086.s007.tif]

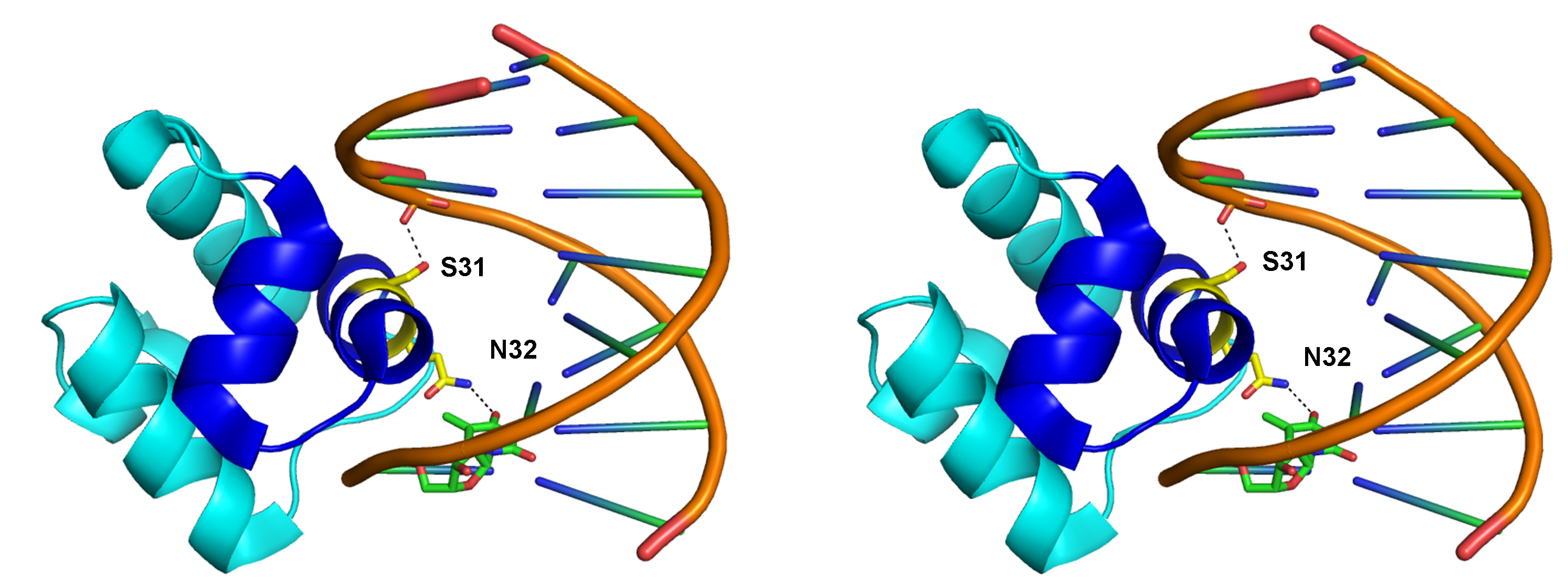

Supplement: S7 Fig — Coloring as in S3 Fig. (TIF) [file pone.0139086.s008.tif]

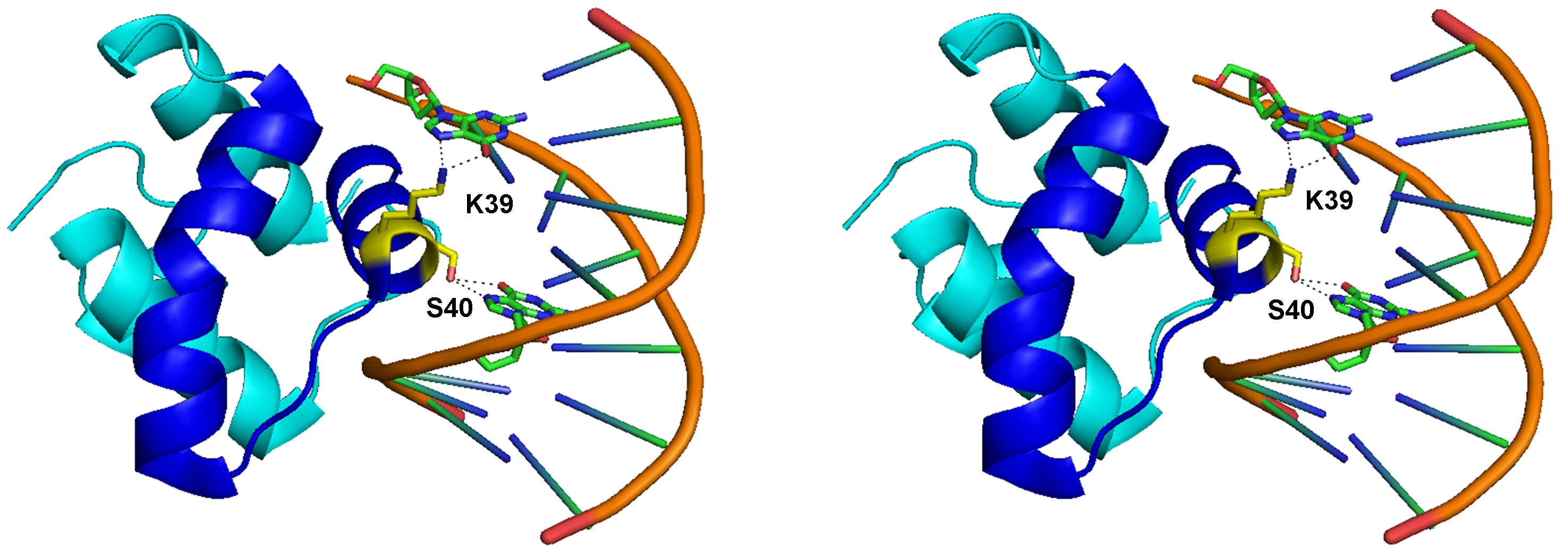

Supplement: S8 Fig — Coloring as in S3 Fig. (TIF) [file pone.0139086.s009.tif]

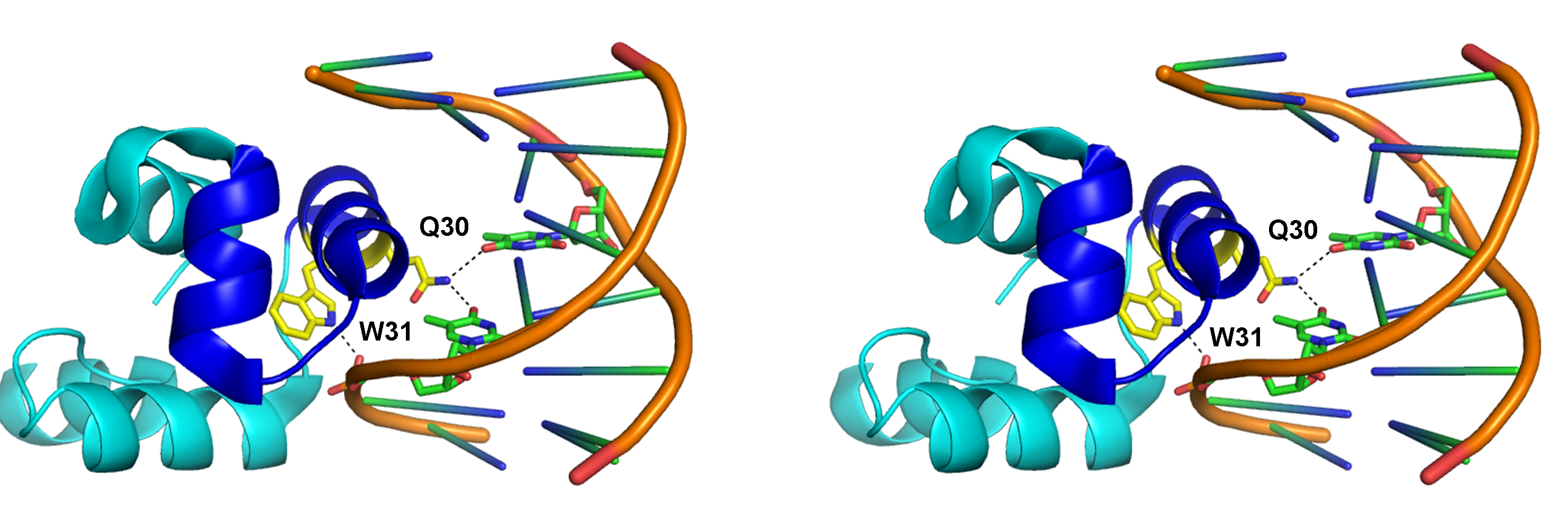

Supplement: S9 Fig — (TIF) [file pone.0139086.s010.tif]

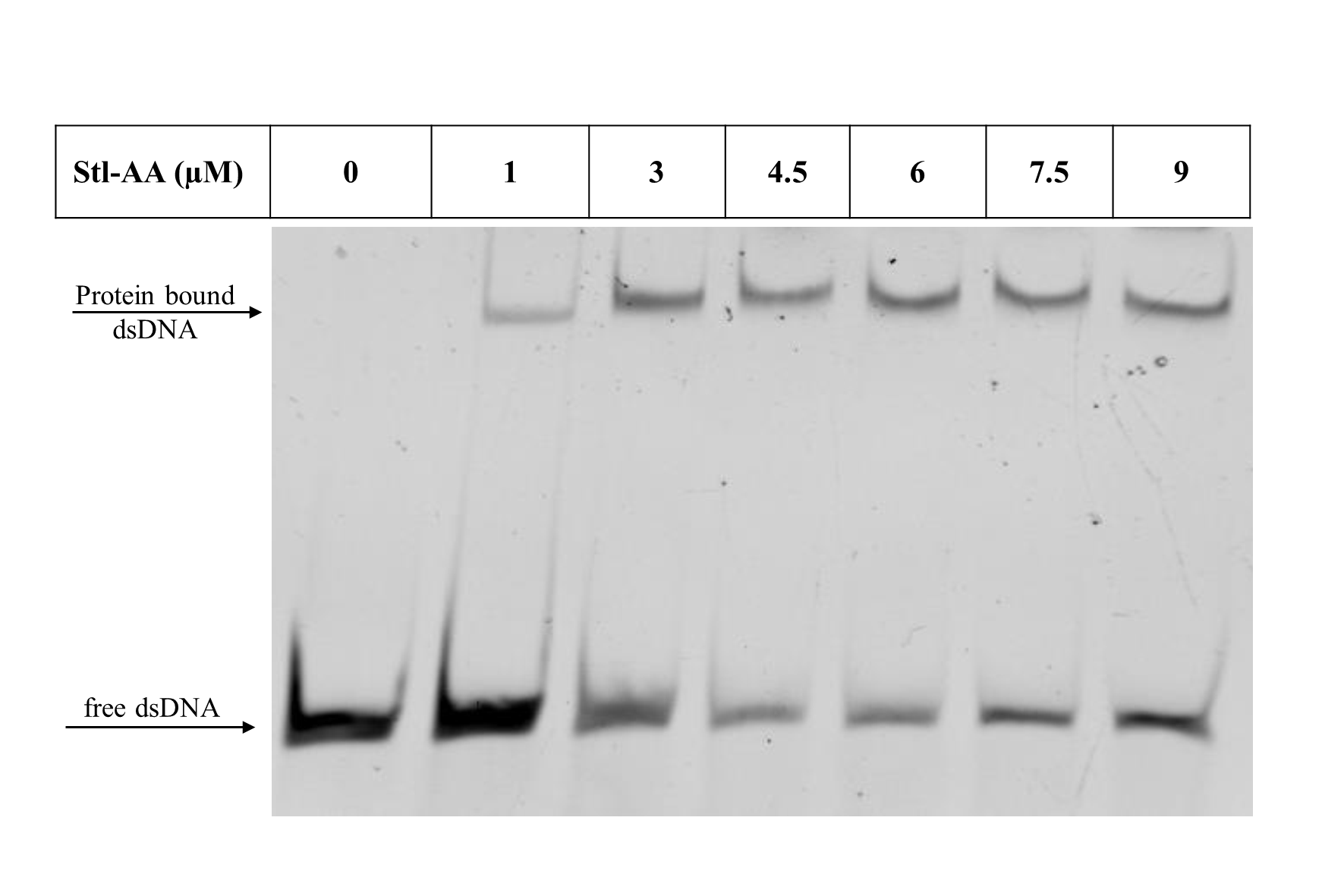

Supplement: S10 Fig — Electrophoretic mobility shift assay was performed to investigate the DNA binding ability of Stl-AA. Species and concentrations given in monomers are indicated on the figure. The band of the dsDNA is only partially sifted upwards even if high concentrations of Stl-AA. Wild type Stl shows shift of the same amount of DNA at concentration of 1μM (cf. Fig 3). (TIF) [file pone.0139086.s011.tif]

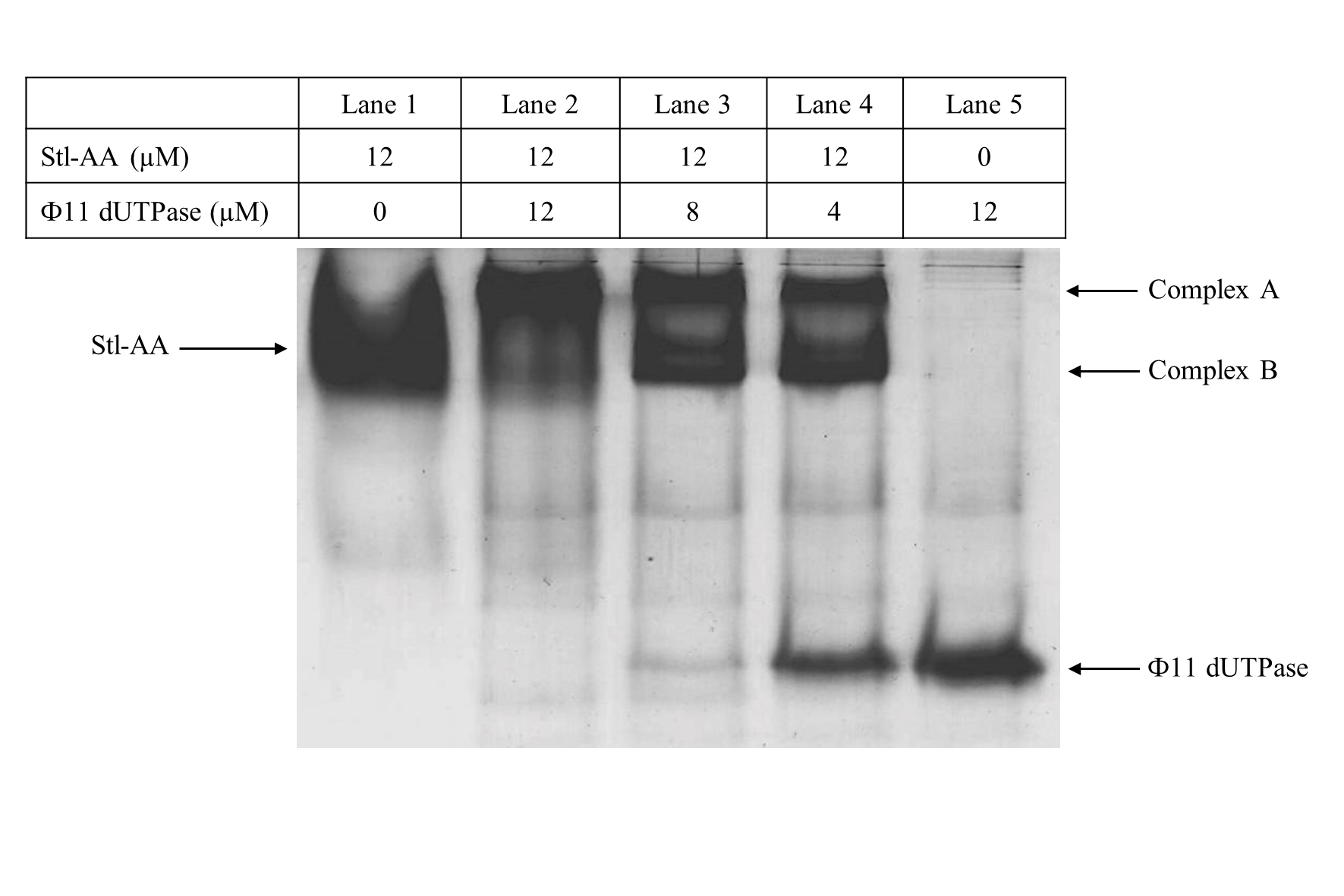

Supplement: S11 Fig — Native gel electrophoresis experiment was performed to investigate the Ф11 dUTPase binding ability of Stl-AA. Species and concentrations given in monomers are indicated. Comparing to the wild type Stl the complex formation of Stl-AA with Ф11 dUTPase is not perturbed (cf. Fig 1B. in [10]). (TIF) [file pone.0139086.s012.tif]

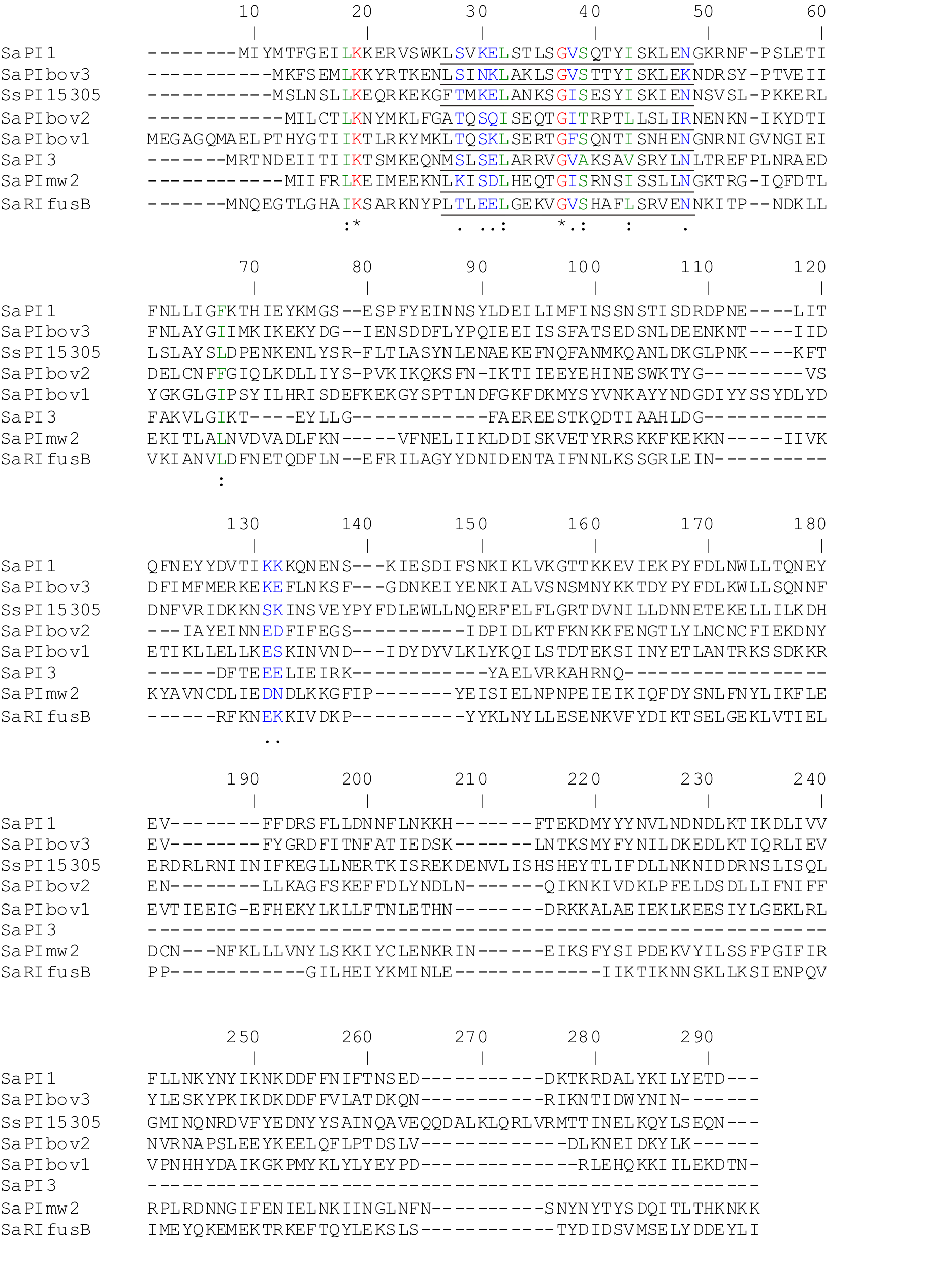

Supplement: S13 Fig — Identical residues are red (*). Strongly similar (:) residues are green, weakly similar residues are blue (.). Residues in the predicted HTH are underlined. (TIF) [file pone.0139086.s014.tif]

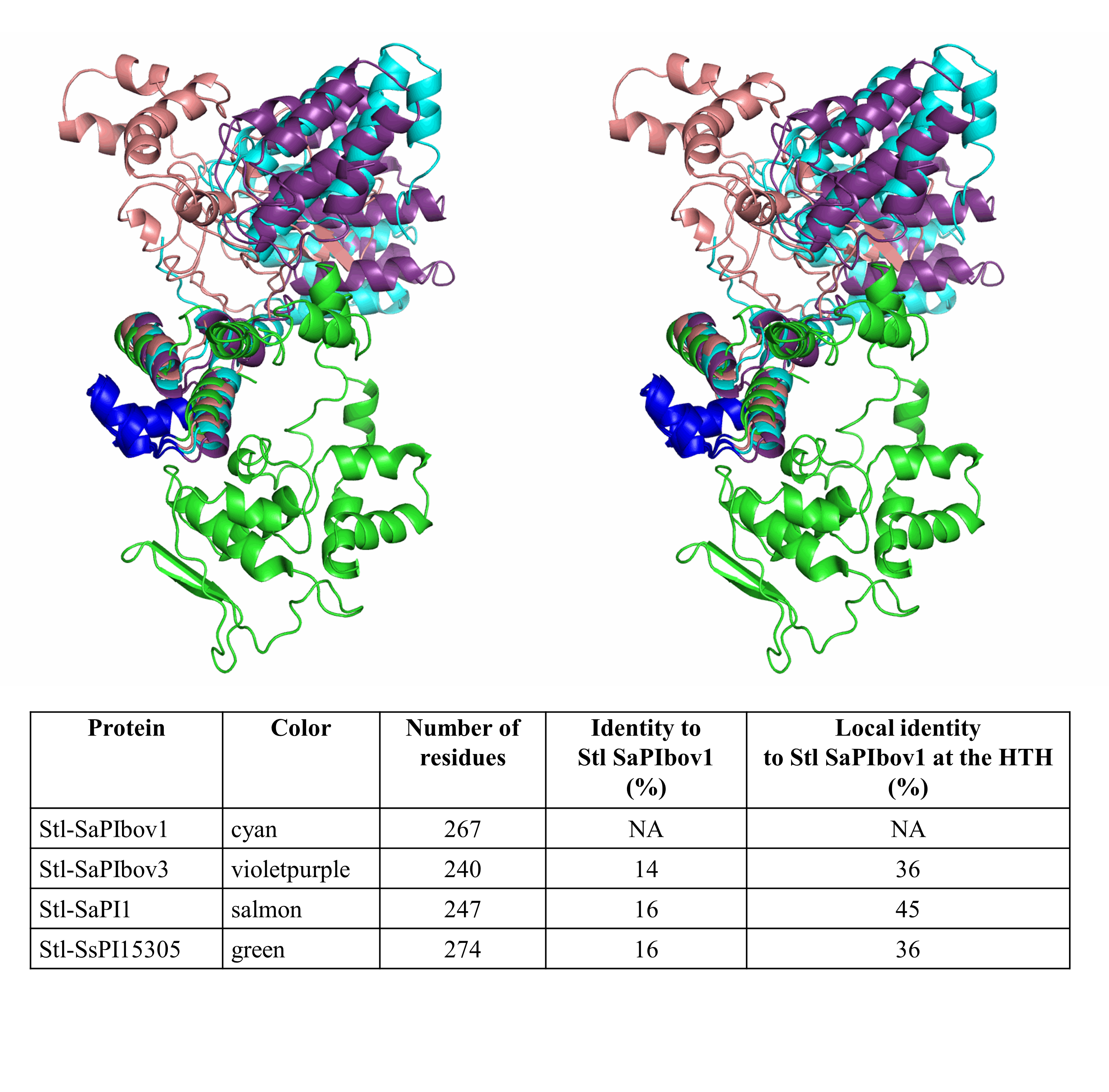

Supplement: S14 Fig — Proteins in cartoon representation. SaPIbov1 Stl is cyan, Stl-like repressors of SaPIbov3, SaPI1, and SsPI15305 pathogenicity islands are violetpurple, salmon, and green, respectively. Predicted HTH motifs of all proteins colored dark blue. (TIF) [file pone.0139086.s015.tif]
